# Rotation of soybean and *Corydalis yanhusuo* enhances yield and active compounds of *C. yanhusuo* via soil nutrient optimisation and rhizosphere microbiome engineering

**DOI:** 10.3389/fpls.2025.1692138

**Published:** 2025-12-09

**Authors:** Jia Liu, Qiang Yuan, Kejie Zhang, Xiaoxiao Sheng, Zixuan Zhu, Ning Sui, Hui Wang

**Affiliations:** 1 Jinhua Academy, School of Pharmaceutical Sciences, Zhejiang Chinese Medical University, Jinhua, China; 2 Qiandao Lake Research Institute, School of Pharmaceutical Sciences, Zhejiang Chinese Medical University, Hangzhou, China

**Keywords:** *Corydalis yanhusuo*, soybean, yield, active component, soil nutrient, microbial community

## Abstract

*Corydalis yanhusuo* W.T. Wang, a herb in the Papaveraceae family used for pain treatment, faces challenges with continuous cropping. Crop rotation with soybean can mitigate soil issues and facilitate the development of subsequent crops. This study evaluated how varying durations of soybean–*C. yanhusuo* rotation affect yield and active component of *C. yanhusuo*, soil nutrients, and microbial communities. Rotation with soybean progressively improved yield and active component of *C. yanhusuo*. Concurrently, soil organic matter, total/hydrolysable nitrogen, and soil enzyme activities improved over time. Microbial OTUs increased in the bulk soil, rhizosphere soil, and roots, along with significant improvements in α-diversity. Over time, the Proteobacteria and pathogenic genera decreased, while Firmicutes and other beneficial genera increased. Network complexity and functions related to nitrate denitrification, cellulolysis, and xylanolysis improved with increased rotation duration. Significant positive correlations were detected between *Bacillus*, *Mortierella*, *Trichoderma*, and yield, medicinal components in *C. yanhusuo*, and soil nutrients. Structural equation modelling revealed that crop rotation affects *C. yanhusuo* yield by influencing the microbial community, which in turn alters soil nutrients. The soybean–*C. yanhusuo* rotation system enhances *C. yanhusuo* yield and active component content by improving soil nutrients and microbial diversity, providing valuable insights for sustainable medicinal plant cultivation.

## 1 Introduction

Monoculture systems are the mainstream cultivation method for global staple crops owing to their advantages in standardised management and compatibility with mechanised operations. This method has been adopted for its simplicity and short-term economic benefits; however, it poses significant challenges to soil health, biodiversity, and long-term agricultural sustainability (Dalzotto Artuzo et al., 2025; Takola et al., 2023). It is essential to implement a diverse array of sustainable agricultural techniques, such as crop rotation, to preserve soil fertility and guarantee long-term agricultural productivity (Waha et al., 2018).

Crop rotation is an efficacious agricultural technique that entails cultivating several crops in a systematic order on the same parcel of land (Gahagan et al., 2023). This method can enhance soil health, increase crop yields, and reduce pest and disease pressure (Yang et al., 2024). Among the diverse rotation combinations, legume crop rotation models demonstrate unique advantages and immense potential (Sauvadet et al., 2021). Soybeans, a typical nitrogen-fixing legume crop, have a symbiotic system of root secretions and rhizobia that can significantly enhance soil nitrogen availability and reconstruct the structure of the soil microbial community through biological nitrogen fixation and root allelopathy (Te et al., 2022). For instance, soybean–maize rotation can increase total nitrogen content in the soil, particularly in the top layers, compared to continuous cropping (Yan et al., 2024). In wheat–legume rotations, leguminous plants enrich the soil with nitrogen, benefiting subsequent wheat crops (Regassa et al., 2023; Yu et al., 2024). Soybean rotation promotes the accumulation of soil organic carbon (Meng et al., 2025). A field experiment lasting eight years in Northeast China revealed that soil carbon and nitrogen stocks were much greater under a soybean-maize rotation compared to continuous farming (Luo et al., 2024). The diverse root systems of soybeans and other crops in rotation help break up soil compaction, increase water infiltration, and reduce soil erosion (Wang et al., 2025). Soybean–maize rotation enhances soil ecosystem multifunctionality in comparison to continuous farming (Carver et al., 2022).

Rotation enhances soil microbial communities and biodiversity. Soybean–maize rotations significantly enhance the number and diversity of soil bacteria, resulting in more complex bacterial community structures, particularly raising specific species (Araujo et al., 2023; Meier et al., 2021). Other beneficial microbes, such as *Bradyrhizobium*, *Gemmatimonas* and *Mortierella*, are more abundant in soils under soybean rotation, contributing to improved soil health and fertility (Tago et al., 2011). Crop rotation efficiently controls soil–borne illnesses and pests by interrupting their life cycles, hence diminishing infestation rates and disease prevalence. For example, cotton–legume rotations significantly lower the occurrence of cotton *Fusarium* wilt and *Verticillium* wilt (Li et al., 2017). Maize–legume rotations can significantly diminish the prevalence of harmful microorganisms, including *Fusarium oxysporum* (Nemadodzi et al., 2023). Well-designed crop rotations optimise nutrient utilisation, minimise deficiencies, and enable crops to reach their full productivity potential. The formation of a positive cycle through a mutual feedback network between nutrients and microorganisms provides a scientific basis for the realisation of sustainable and healthy soil remediation in crop rotation systems (Zverev et al., 2023). Crop rotation has proven effective for staple crops; however, its application to medicinal plants remains understudied.

*Corydalis yanhusuo* is a valuable resource in Chinese herbal medicine, characterised by a complex chemical composition that contains various active components. Benzylisoquinoline alkaloids, such as tetrahydropalmatine, protopine, and aporphine, are the primary active components and have significant analgesic effects (Xu et al., 2021). With the continuous growth of the market demand for *C. yanhusuo*, its cultivation area has expanded rapidly. The shallow root structure and high nutrient consumption of *C. yanhusuo*, a typical rhizome medicinal plant (Chen et al., 2020; Zhao et al., 2023), increase the risk of soil degradation in continuous cropping. *Corydalis yanhusuo* is mainly cultivated in long-term, continuous monocultures, which have led to a series of serious problems. Continuous cultivation of *C. yanhusuo* modifies soil microbial populations and degrades soil structure, jeopardising the sustainability of its production (Li et al., 2021).

To address soil degradation caused by the continuous cropping of *C. yanhusuo*, a crop rotation system incorporating soybeans and *C. yanhusuo* has been proposed. The optimal planting period for *C. yanhusuo* is from September to mid–October, with harvesting occurring in May (Li et al., 2021). Sowing soybeans in June of the subsequent year can increase the land utilisation rate. However, the specific effects and underlying mechanisms of soybean-*C. yanhusuo* rotation systems on soil health and microbial dynamics remain unclear, necessitating systematic investigation to validate and optimise this agronomic strategy.

This study examined the impact of rotation systems involving soybean and *C. yanhusuo*, and established the following treatments: mono–crop cultivation of *C. yanhusuo* (C1), a one–year rotation of soybean and *C. yanhusuo* (SC1), and a two–year rotation of soybean and *C. yanhusuo* (SC2). High-throughput sequencing, analysis of soil physicochemical properties, and structural equation modelling were adopted to analyse variations in soil physicochemical properties across different systems, as well as the characteristics of rhizosphere microbial communities and their synergistic regulatory effects on the growth of *C. yanhusuo*. Our research aims to: (1) evaluate the effects of the different duration of soybean and *C. yanhusuo* rotations on the yield and active component content of *C. yanhusuo*; (2) clarify the effects of the different duration of soybean and *C. yanhusuo* rotations on the physicochemical properties of the soil and microbial community in bulk soil, rhizosphere soil, and roots; (3) analyse the co-occurrence network and functional changes of soil microorganisms in the different duration of soybean and *C. yanhusuo* rotations. Our study provides a theoretical basis for optimising crop rotation systems for medicinal plants.

## 2 Materials and methods

### 2.1 Plant materials and field experiment

**Figure 1:**
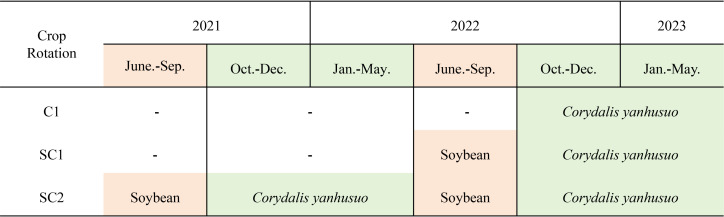
Rotation scheme of soybean with *C. yanhusuo*.

The seeds of *C. yanhusuo* were collected in Dongyang City, Zhejiang Province, and authenticated by Professor Shuili Zhang of Zhejiang University of Traditional Chinese Medicine as *C. yanhusuo*. *Corydalis yanhusuo* has been included in the Chinese Field Herbarium, in which the specimen information of *C. yanhusuo* (PE 01040942) has been explicitly documented. *Corydalis yanhusuo* is a herbaceous plant belonging to Plantae, Tracheophyta, Magnoliopsida, Ranunculales, Papaveraceae, and *Corydalis* genus. The field trial was carried out in Dongyang City, Zhejiang Province (29°16′N, 116°36′E), at an altitude of 265 meters. Before the experiment, a comprehensive analysis of both soil physical and chemical properties was conducted to establish baseline conditions (**Supplementary Tables 1**, **2**). The soil bulk density (BD) was 1.18 g/cm^3^, indicating a loose to moderately compacted state that allows for root penetration and growth. Total porosity was 55.47%, which supports both rapid drainage and adequate water retention. Other soil chemical properties were: pH 6.50, soil organic matter (SOM) 12.55 g/kg, total nitrogen (TN) 1.66 g/kg, total phosphorus (TP) 0.64 g/kg, total potassium (TK) 7.33 g/kg, hydrolysable nitrogen (HN) 210.53 mg/kg, available phosphorus (Ava-P) 45.37 mg/kg, and available potassium (Ava-K) 365.55 mg/kg. The experiment took place between June 2021 and May 2023, utilising a randomised block design approach. The experimental site was partitioned into three distinct plots, each measuring 5×5 m^2^. The treatments were categorised into three groups, with three replicates per group: (1) control group (C1), monoculture of *C. yanhusuo*. For C1 plots, the three individual plots were left fallow (i.e., no crops were planted) during the last growing season; (2) one-year rotation (SC1): *C. yanhusuo* were cultivated for one year on plots that had previously been used for soybean cultivation in the preceding growing season; (3) two-year rotation group (SC2): the same planting method as that in SC1 was adopted, with repeated cultivation in an additional year. The cultivation periods for *C. yanhusuo* and soybean are illustrated in **Figure 1**. The plots assigned to these three treatment methods were situated next to one another and were subjected to identical cultivation management approaches.

### 2.2 Collection of bulk soil, rhizosphere soil, and root samples

At each of the four corners and the central point of every experimental plot, 15 individual plants were randomly selected to serve as biological replicate samples. Soil samples were collected at a depth of 10–20 cm. Soil samples from both the bulk and rhizosphere zones were gathered in accordance with the procedure described (Herrera et al., 2015). Plants were gently held and their root systems lightly shaken to allow loosely adhering soil on the root surface to naturally fall off. The soil tightly attached to the roots was carefully brushed away and designated as rhizosphere soil; it was then stored in sterilised bags. Bulk soil samples were gathered in close proximity to the roots and similarly placed in sterilised bags for subsequent analysis. As per Herrera et al., collected plant root samples were thoroughly rinsed with tap and distilled water to remove soil particles. The gathered tissue samples were first submerged in 75% ethanol for 5 min, rinsed with 3% sodium hypochlorite solution for 3.5 min, briefly immersed again in 75% ethanol for 35 s.

### 2.3 Assessment of soil physical and characteristics and enzymatic activities

Soil physical properties were calculated as follows: Undisturbed original soil samples were collected using a cutting ring with a specific volume. Once the cutting ring was filled with the soil sample, the sample was transferred to an aluminium box with a known mass. Subsequently, the aluminium box containing the soil sample was placed in an oven and baked at 105 °C for 4 h. Then, it was cooled in a desiccator and weighed again. The soil BD (g/cm^3^) was calculated using the following formula:


$$\text{BD}=\frac{{\text{M}}_{2}-{\text{M}}_{1}}{\text{V}}$$

M_1_ is the mass (g) of the empty aluminium box, M_2_ is the mass (g) of the aluminium box containing the dried soil sample, and V is the volume (cm^3^) of the cutting ring.

Soil porosity refers to the percentage of pore volume within the soil to the total soil volume under natural conditions. The formula for calculating soil porosity (P) is as follows:


$$\text{P}=1-\frac{\text{BD}}{\text{soil particle density}}$$

where the soil particle density is calculated using an average value of 2.65 g/cm^3^.

The analytical techniques employed for assessing soil physicochemical properties adhered to established methodologies from classical literature (Bulluck et al., 2002). pH value was determined using a pH meter, with measurements taken from a soil suspension prepared by mixing soil with 0.01 M CaCl_2_ solution at a ratio of 1:2.5 (w/v). SOM was quantified using the K_2_CrO_7_ oxidation titration method. TN was determined using an elemental analyser, following the principles of the Kjeldahl method. Soil was digested by H_2_SO_4_/HClO_4_, and TP was measured using the molybdenum blue method. TK was determined by flame photometry after digesting the soil samples in aqua regia. HN, Ava-P, Ava-K, soil urease (S-UE), soil sucrase (S-SC) and soil acid phosphatase (S-ACP) were determined using assay kits manufactured by Solarbio Science & Technology Co., Ltd., Beijing, China. All soil measurement data and statistical analysis data can be found in the attachments (**Supplementary Tables 1**-**35**).

### 2.4 Extraction of DNA from soil and sequencing

Genomic DNA from microbes was extracted from the samples using the procedure included with the Omega Bio-tek soil DNA extraction kit (Norcross, GA, USA). PCR amplification was conducted utilising specific primer sets: 799F/1193R for the bacterial 16S rRNA gene V4–V5 hypervariable region, and ITS1F/ITS1R for the fungal internal transcribed spacer (ITS1) region (**Supplementary Table 36**). The PCR reaction mixture comprised 0.8 μL each of forward and reverse primers, 4 μL of 5× FastPfu buffer, 0.4 μL of FastPfu DNA polymerase (TransGen Biotech Co., Ltd., Beijing, China), 2 μL of 2.5 mM dNTPs, and 20 ng of genomic DNA template.

PCR amplification was carried out as follows: 95°C for 7 min, followed by 32 cycles of denaturation at 95°C for 35 s, primer annealing at 58°C for 45 s, and strand extension at 72°C for 45 s, a final extension at 72°C for 5 min, after which the samples were cooled to 10°C to halt the reaction. Each sample underwent three independent technical replicates during PCR amplification. Following gel electrophoresis, the target amplification products were excised and purified from the gel matrix. Sequencing was done by Majorbio Bio-Pharm Technology Co. Ltd. in Shanghai, China. The raw sequencing reads (SRR33853140 to SRR33853193) are deposited in the NCBI Short Read Archive.

### 2.5 Data processing

A custom Perl script was employed to demultiplex the raw FASTQ files, and fastp (version 0.19.6) was used to filter the files for quality (Chen et al., 2018). FLASH (version 1.2.7) was then used to combine the filtered reads and create continuous sequences (Magoč and Salzberg, 2011). UPARSE version 7.1 was used to categorise the filtered and optimised sequences into operational taxonomic units (OTUs) (Edgar, 2013). Rarefaction curves and alpha diversity indices were computed using the OTU clustering results in Mothur (version 1.30.1) (Schloss et al., 2009). Microbial community compositional resemblance across samples was evaluated using principal coordinate analysis (PCoA) with Bray-Curtis dissimilarity metrics, performed via the Vegan package version 2.5-3. Linear discriminant analysis (LDA) effect size was conducted to identify bacterial and fungal taxa that were significantly more abundant in the different samples (LDA score > 2, *P* < 0.05) (Segata et al., 2011). Bacterial functional profiles were predicted using FAPROTAX based on OTU data, while fungal communities were functionally annotated through the FUNGuild database (Langille et al., 2013; Tanunchai et al., 2023). The Majorbio platform (https://cloud.majorbio.com/) was used to calculate correlations, with R > 0.5 and *P* < 0.05. Gephi (v0.9.2) was used for visualisation. Structural Equation Models (SEM) simultaneously consider relationships among multiple variables. Upon attaining a satisfactory model fit for microbial diversity, the path coefficients of the model and their associated *P*-values are analysed. SEMs were parameterised using our dataset and fit to adhere to a Chi-square test *P*-value > 0.05 and a Fish’s C test *P*-value > 0.05 to ensure that the data were normally distributed.

### 2.6 Determination of tetrahydropalmatine and protopine of *C. yanhusuo*

For tetrahydropalmatine and protopine extraction, 0.5 g of dried tuber powder from each sample was combined with 20 millilitres of a 20:1 v/v methanol-ammonia solution and subjected to ultrasonic-assisted extraction (1 h, room temperature). The resulting extracts were clarified through Millipore filtration and stored at 4°C prior to quantification via high-performance liquid chromatography (HPLC). Tetrahydropalmatine and protopine standard solutions were prepared by precisely weighing reference standards (Shanghai Yuanye Bio-Technology Co., Ltd.) and dissolving them in an appropriate solvent to create stock solutions at concentrations of 46 and 35 µg/mL, respectively. The HPLC system used in this experiment consisted of a 2998 PDA detector and an e2695 separation module (Waters Corporation, Milford, USA). HPLC experiments were performed on a C18 column. The HPLC separation utilised a binary mobile phase comprising eluent A (acetonitrile) and eluent B (aqueous solution containing 0.1% phosphoric acid, with pH adjusted to 6.0 using triethylamine).

### 2.7 Statistical analysis

Statistical analysis of all datasets was conducted using IBM SPSS Statistics version 24.0 (SPSS Inc., Chicago, IL, USA). Data normality was assessed using the Shapiro-Wilk test, and homogeneity of variances was evaluated using Levene’s test prior to analysis of variance (ANOVA). One-way ANOVA was performed to analyse differences among treatment groups. Means were compared using Tukey’s Honestly Significant Difference (HSD) test when ANOVA revealed significant differences (*P* < 0.05). Data are presented as mean values ± standard deviation (SD).

## 3 Results

### 3.1 Impact of yield and the concentration of active compounds of *C. yanhusuo* under soybean–*C. yanhusuo* rotation

**Figure 2:**
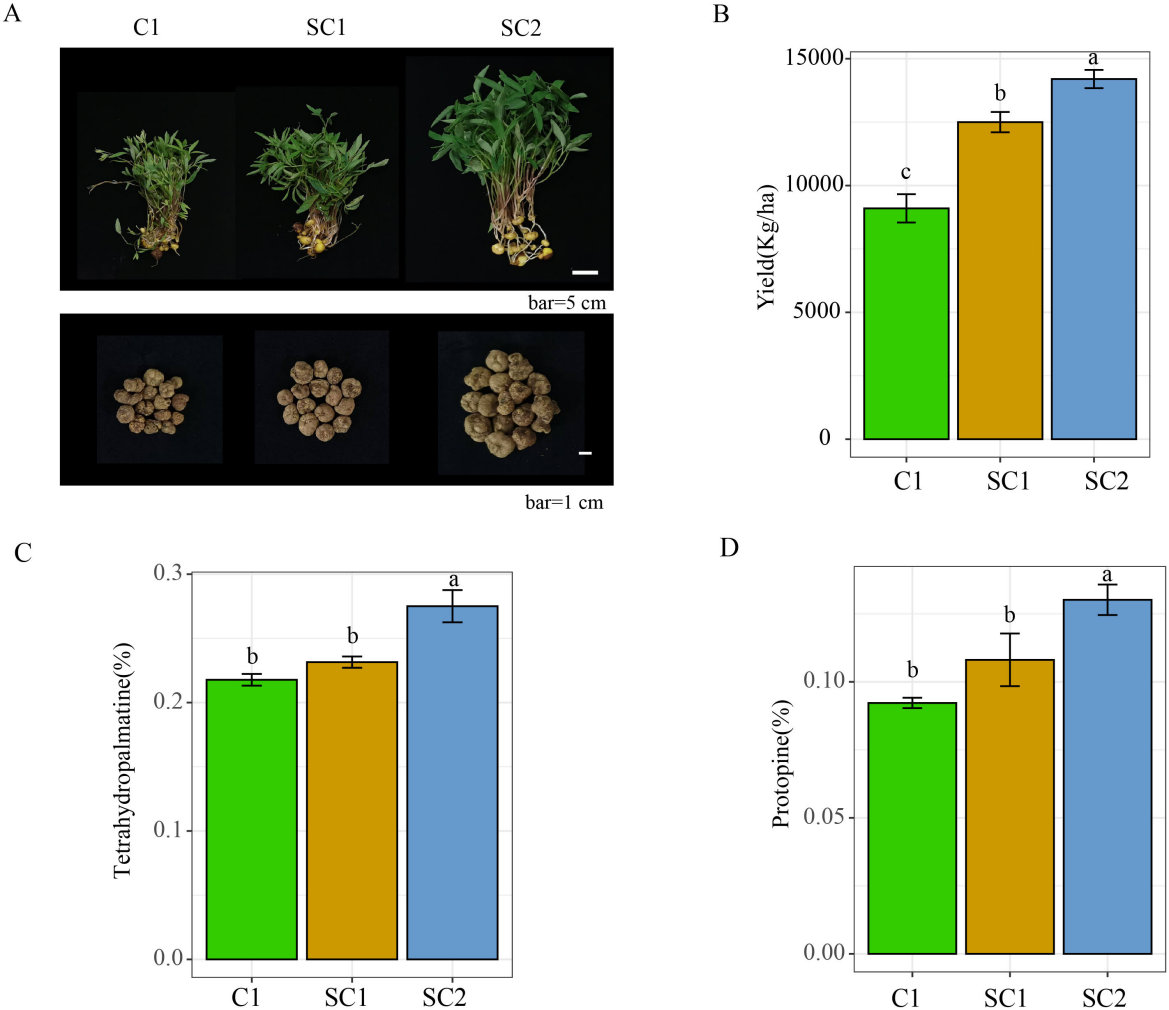
Images of *C. yanhusuo* from the three distinct treatments **(A)**. **(B)** Yield of *C. yanhusuo* tubers. Content of tetrahydropalmatine **(C)** and protopine **(D)** in *C. yanhusuo*. Distinct lowercase letters signify substantial variations among various samples (*P* < 0.05). C1, monoculture of *C*. *yanhusuo*; SC1, soybean and *C. yanhusuo* rotation for one year; SC2, soybean and *C. yanhusuo* rotation for two years. -, no crops planted.

We gathered *C. yanhusuo* from soils under the monoculture of *C. yanhusuo* (C1), after the rotation of soybeans and *C. yanhusuo* for one year (SC1) and two years (SC2) cultivation conditions. Tubers of *C. yanhusuo* in SC1 and SC2 were large, plump, and solid, while those in C1 were smaller (**Figure 2A**). The total yield of *C. yanhusuo* tubers in C1 was 9,100 kg/ha, while those in SC1 and SC2 were 12,500 and 14,200 kg/ha, respectively (**Figure 2B**, **Supplementary Tables 37**-**45**). The principal components of *C. yanhusuo* are tetrahydropalmatine and protopine. Compared with C1, the content of tetrahydropalmatine and protopine in *C. yanhusuo* in SC1 and SC2 significantly increased (**Figures 2C, D**) by 6.29% and 26.69% and by 17.16% and 40.97%, respectively. Soybean–*C. yanhusuo* rotation thus improved the quality of *C. yanhusuo*, especially in SC2.

### 3.2 Soybean–*C. yanhusuo* rotation boosts soil nutrients and enzyme activity

Compared with C1, the soil pH values in SC1 and SC2 increased. However, pH values did not significantly differ between SC1 and SC2 (**Table 1**). Soil pH before treatment was slightly higher than that in C1 and equal to that in SC1. As the number of years of soybean-*C. yanhusuo* rotation increased, the contents of HN, TN, SOM, Ava-P, and Ava-K in the gradually increased. Specifically, the HN content in SC2 was significantly higher than that in C1 and SC1. Before treatment, the soil HN content was slightly higher than that in C1, while C1 had the lowest HN content. The TN content was the highest in SC2, with no significant differences among SC1 and C1. The contents of SOM, Ava-P, and Ava-K were the highest in SC2, followed by SC1, while no significant differences were observed between SC1 and SC2. Before treatment, the contents of SOM, Ava-P, and Ava-K in the soil were slightly higher than those in C1, but without being significantly different. Moreover, TP was the highest in SC2, followed by SC1. TK did not significantly differ among C1, SC1, and SC2 or when comparing these to the state before treatment.

**Table 1:** Effect of soybean–*C. yanhusuo* rotation on physical and chemical properties of soil and enzyme activities.

Treatments	Before treatment	C1	SC1	SC2
pH	6.50±0.05a	5.67±0.35b	6.60±0.36a	7.03±0.25a
SOM(g/kg)	12.55±0.96b	12.17±1.01b	14.57±1.62ab	16.44±0.95a
TN(g/kg)	1.66±0.01c	1.68±0.01bc	1.69±0.01b	1.76±0.006a
TP(g/kg)	0.64±0.02b	0.65±0.01ab	0.69±0.03ab	0.70±0.02a
TK(g/kg)	7.33±0.09a	7.13±0.03a	7.28±0.14a	7.36±0.10a
HN(mg/kg)	210.53±2.80c	196.00±1.40d	223.07±2.14b	246.87±4.28a
Ava-P(mg/kg)	45.37±2.69b	43.26±1.66b	50.64±0.94a	52.68±1.95a
Ava-K(mg/kg)	365.56±5.09b	356.67±4.40b	389.72±4.27a	395.77±15.84a
S-SC(U/g)	4.44±0.09c	4.49±0.21c	5.16±0.11b	5.69±0.12a
S-UE(U/g)	234.79±15.03c	244.22±6.88c	303.54±8.12b	390.05±14.75a
S-ACP(U/g)	570.77±31.02c	569.60±25.86c	733.11±27.37b	931.59±31.74a
BD(g/cm^3^)	1.18±0.02a	1.21±0.02a	1.17±0.021a	1.16±0.026a
Porosity(%)	55.47±0.76a	54.21±0.58a	55.97±0.79a	56.22±0.1a

Ava-P, available phosphorus; Ava-K, available potassium; HN, hydrolysable nitrogen; SOM, soil organic matter; TN, total nitrogen; TP, total phosphorous; TK, total potassium; S-SC, soil sucrase; S-UE, soil urease; S-ACP, soil acid phosphatase; BD, soil bulk density. Distinct lowercase letters signify substantial variations among various samples (*P* < 0.05).

The activities of the soil enzymes S-SC, S-UE, and S-ACP were increased in SC1 and SC2 compared with those in C1. The activities of S-SC, S-UE, and S-ACP reached the highest levels in SC2 (5.69, 390.05, and 931.59 U/g, respectively; **Table 1**). Before treatment, the activities of S-SC and S-UE were slightly lower than those in C1 but the difference was not significant.

Comparison of soil physical parameters indicated that soil bulk density in C1 increased slightly compared to that before treatment. The bulk density in SC2 was lower than that in SC1, and that in SC1 was lower than that before treatment, but this difference was not significant. The soil porosity exhibited an opposite trend.

### 3.3 Effect of soybean–*C. yanhusuo* rotation on bulk soil, rhizosphere soil, and root microbial community characteristics

To investigate the microbiome in bulk soil, rhizosphere soil, and root systems of soybean and *C. yanhusuo* throughout different rotation years, 27 samples were sequenced. Amplicon sequencing yielded 1,815,904 reads of the bacterial 16S rRNA region and 2,341,445 reads of the fungal ITS region (**Supplementary Table 46**). Rarefaction curve analysis demonstrates a swift initial rise in observed OTU counts, subsequently reaching a plateau, signifying that the sequencing depth has attained adequate coverage to encompass the majority of microbial diversity inside the samples (**Supplementary Figure 1**). The OTUs in the bacterial and fungal communities of SC2 were considerably greater than those in groups SC1 and C1 (**Supplementary Figure 2**). Bulk soil bacterial OTUs, rhizosphere soil bacterial OTUs, and root fungal OTUs of SC1 group were comparable to those of the C1 group; however, in other groups, the OTU counts of SC1 were considerably greater than those of group C1 (**Supplementary Figure 2**).

**Figure 3:**
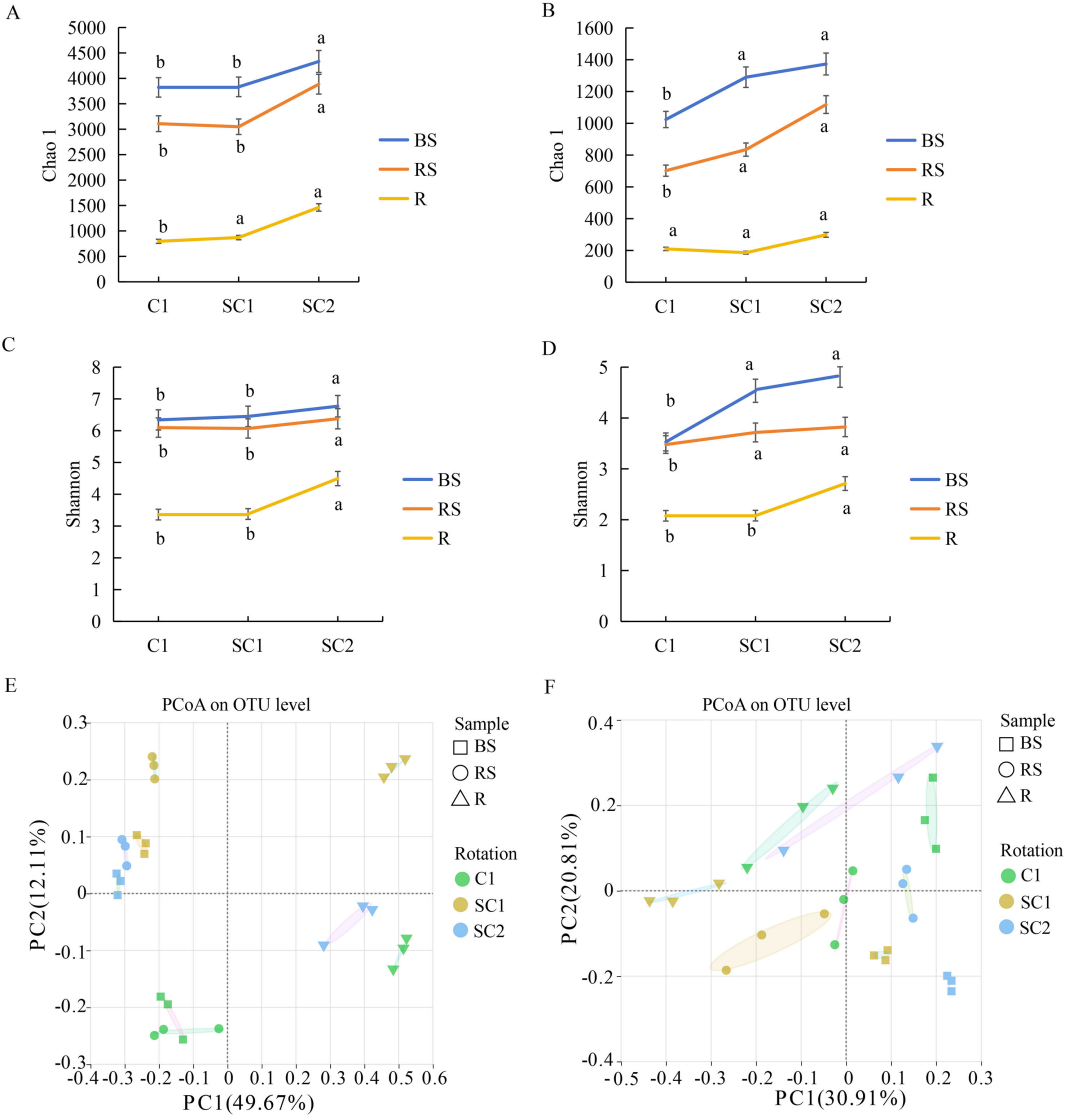
Influence of cropping practices on soil communities. The Chao-1 index at the OTU level: **(A)** Bacteria, **(B)** Fungi. The Shannon index at the OTU level: **(C)** Bacteria, **(D)** Fungi. PCoA analysis at the OTU level: **(E)** Bacteria, **(F)** Fungi. Distinct lowercase letters signify substantial variations among various samples (*P* < 0.05).

The Chao-1 index indicates microbial richness, while the Shannon index reflects microbial diversity. A greater Chao-1 index and Shannon index indicate increased community richness and diversity. At the bacterial level, the Chao-1 index in bulk soil in SC2 was substantially higher (4,332) than that in SC1 (3,833) and C1 (3,823). The Chao-1 index in the rhizosphere soil of SC2 was the highest (3,885), followed by SC1 and C1. The Chao-1 index in the root of SC2 and SC1 was noticeably higher than that of C1 (**Figure 3A**). Similarly, at the fungal level, the Chao-1 in the bulk soil and rhizosphere soil of SC1 and SC2 was abundantly higher than that of C1. The Chao-1 index in the root of SC2 was higher than that of C1, without differences (**Figure 3B**). Summarily, with the extension of the soybean and *C. yanhusuo* rotation period, the bacterial and fungal richness in bulk soil, rhizosphere soil, and root all showed an increasing trend. Regardless of the bacterial diversity in bulk soil, rhizosphere soil, or root, the Shannon index of SC2 was the highest, followed by SC1; C1 was the lowest. There were sizeable differences between the SC2 group and the C1 group. Although the SC1 group increased compared to the C1 group, there was no significant difference (**Figure 3C**). At the fungal level, the Shannon index in the bulk soil and rhizosphere soil of SC1 and SC2 was considerably higher than that of C1. Although no noticeable difference was seen, the Shannon index in the roots of SC1 exceeded that of C1, and the Shannon index in the roots of SC2 was markedly greater than that of C1 (**Figure 3D**). With the increase in years of soybean rotation, bacterial and fungal diversity in the bulk soil, rhizosphere soil, and root all exhibit a striking increasing trend. Conclusively, the cultivation method involving crop rotation between soybean and *C. yanhusuo*, along with an extended duration of rotation, markedly influences the richness and diversity of rhizosphere microorganisms.

PCoA was employed to visualise and interpret the compositional differentiation of microbial communities across experimental treatments (**Figures 3E, F**). Based on the results, the samples can be divided into 9 groups. The microbial community structure varies significantly among different treatments, indicating that rotation between soybean and *C. yanhusuo* has a pronounced impact on the composition of the microbial community.

### 3.4 Effect of soybean–*C. yanhusuo* rotation on bulk soil, rhizosphere soil, and root microbial community compositions

**Figure 4:**
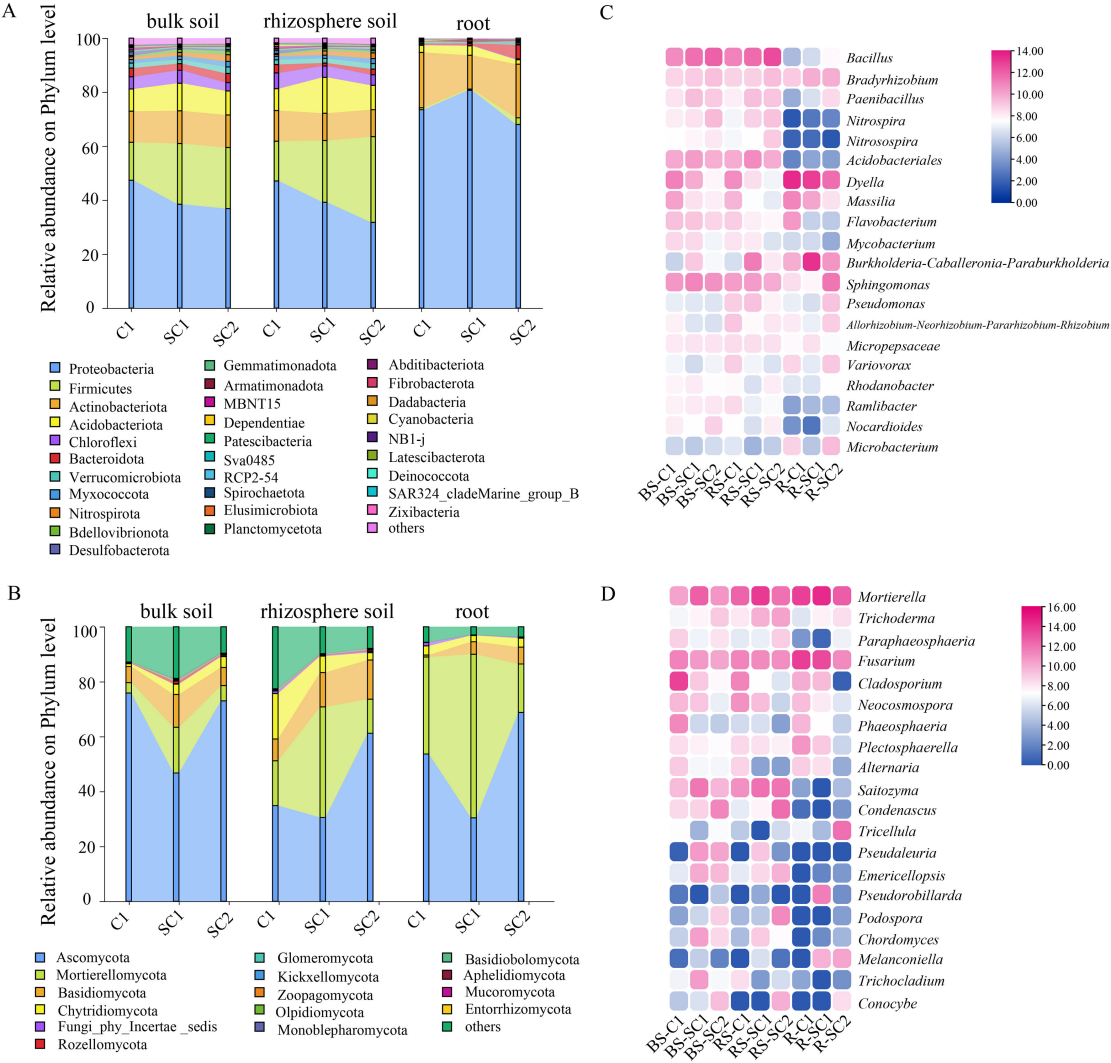
Effect of soybean–*C. yanhusuo* rotation on microbial community compositions. Percent of the bacterial community abundance **(A)** and fungi community abundance **(B)** at the phylum level. Heatmap depicting the relative abundance of **(C)** bacteria and **(D)** fungi at the genus level.

The bacteria and fungi in bulk soil, rhizosphere soil, and roots under different treatments were analysed at the phylum and genus classification levels. The bacterial microbial communities of different treatments mainly consist of the bacterial phyla Proteobacteria (31.74–80.67%), Firmicutes (0.44–31.77%), Actinobacteriota (9.96–20.49%), Acidobacteriota (1.67–13.30%), Chloroflexi (0.16–5.85%) and Bacteroidota (0.46–5.20%) (**Figure 4A**). However, the abundance of the dominant microbial phyla significantly changed following different treatments. Proteobacteria dominated as the predominant bacterial phylum in the bulk soil and rhizosphere soil of C1 treatment. However, one year after soybean rotation (SC1), the microbial community restructuring was characterised by a significant decline in Proteobacteria relative abundance, concurrent with a marked elevation in Firmicutes proportion. Meanwhile, after two years of soybean rotation (SC2), Firmicutes and Proteobacteria exhibited comparable relative abundances, accounting for 31.8% and 31.75% of the bacterial community, respectively. Whether in bulk or rhizosphere soil, Firmicutes showed a striking increasing trend with an extension of the soybean rotation period. The evolving trend of microbial community composition in bulk soil parallels that in rhizosphere soil. The relative abundance of Proteobacteria was the highest (C1-R: 73.50%, SC1-R: 80.67%, SC2-R: 68.0%), followed by Actinobacteriota (C1-R: 20.50%, SC2-R: 12.54%, SC2-R: 19.84%). The relative abundances of Firmicutes and Acidobacteriota were considerably lower than those in soil samples. In the root samples, the relative abundance of Actinobacteriota showed a decreasing trend with an extension of the soybean rotation period. Under different rotation years of *C. yanhusuo*, the dominant fungal phyla were detected as Ascomycota (30.41–75.87%), Mortierellomycota (3.76–59.57%), and Basidiomycota (0.71–14.26%). Chytridiomycota (1.04–16.55%), and Fungi_phy_Incertae_sedis (0.058–1.26%) (**Figure 4B**). The relative abundance of Ascomycota in bulk soil, rhizosphere soil, and root samples initially decreased and subsequently increased following soybean rotation, whereas Mortierellomycota displayed an initial increase followed by a decline.

The dominant genera of bacteria detected in bulk soil were *Dyella*, *Bacillus*, *Burkholderia*-*Caballeronia*-*Paraburkholderia*, *Massilia*, *Bradyrhizobium*, and *Sphingomonas* (**Figure 4C**). With the increase of the rotation years of soybean rotation, the relative abundances of *Bacillus*, *Bradyrhizobium*, *Paenibacillus*, *Nitrospira*, and *Nitrosospira* showed a significant upward trend. Among them, the abundance of *Bacillus* in the rhizosphere soil increased from 6.67% in group C1 to 10.47% in SC1 and 19.71% in SC2. The abundance of *Bradyrhizobium* in the rhizosphere soil increased from 1.52% in group C1 to 2.18% in SC1 and 2.48% in SC2. Furthermore, the relative abundance of *Burkholderia*–*Caballeronia*–*Paraburkholderia* in SC1-R and SC2-R was 47.18% and 32.49%, respectively, which was significantly higher than that in group C1 (29.50%). The relative abundances of *Dyella*, *Massilia*, and *Flavobacterium* in bulk soil and rhizosphere soil gradually decreased. Among them, the abundance of *Dyella* in rhizosphere soil decreased by 91.62% (**Figure 4C**). At the fungal genus level, *Mortierella*, *Fusarium*, *Cladosporium*, *Saitozyma*, *Trichoderma*, and *Paraphacosphaeria*, among others, showed relatively high abundance in bulk soil, rhizosphere soil, and roots. With the increase in crop rotation years of soybean, the relative abundance of *Mortierella*, *Trichoderma*, and *Paraphacosphaeria* in bulk soil, rhizosphere soil, and the roots demonstrated an increasing trend. Among them, the abundance of *Trichoderma* in the rhizosphere soil increased from 0.06% in group C1 to 2.42% in group SC1 and 3.36% in SC2. The relative abundances of *Fusarium*, *Cladosporium*, *Neocosmospora*, *Phaeosphaeria*, *Plectosphaerella*, and *Alternaria* gradually decreased. Among them, the abundance of *Fusarium* in the root decreased from 36.44% in group C1 to 27.07% in group SC1 and 6.00% in SC2. The abundance of *Cladosporium* in the bulk soil decreased from 32.63% in group C1 to 1.35% in group SC1 and 0.44% in SC2 (**Figure 4D**).

### 3.5 Indicator species analysis in bulk soil, rhizosphere soil, and roots under soybean–*C. yanhusuo* rotation

**Figure 5:**
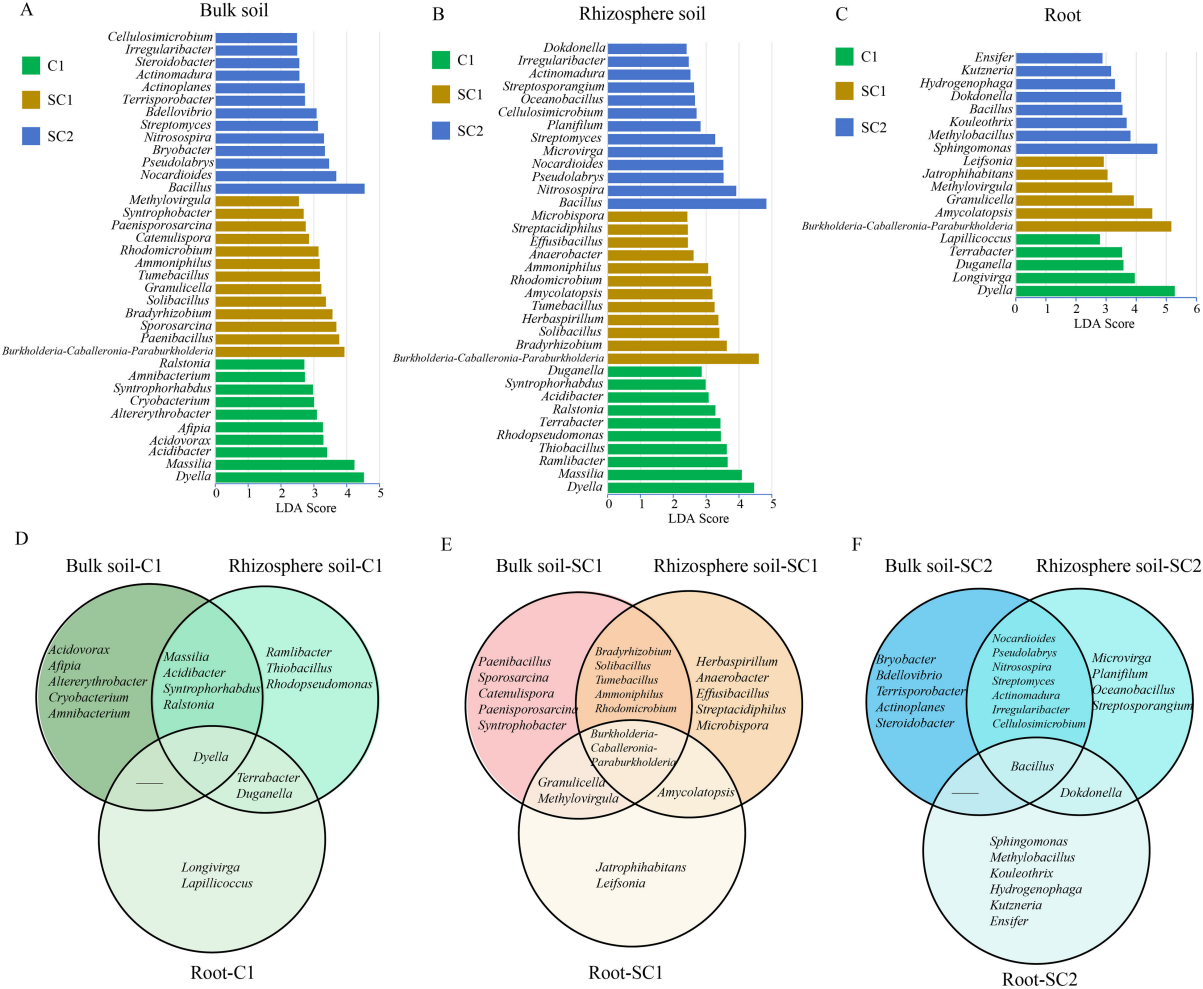
LEfSe analysis of bacterial microbial community differences in different cropping practices. LDA based on the level of bacterial microbial genera showed the microbial classes that caused significant differences between different treatments in bulk soil **(A)**, rhizosphere soil **(B)**, and roots **(C)**. The green, yellow, and blue indicate that the taxa are in the C1, SC1, and SC2 groups, respectively. Veen analysis of bacterial communities in bulk soil, rhizosphere soil and roots in the different cropping systems. Bacterial community in C1 **(D)**, SC1 **(E)**, and SC2 **(F)**.

LDA LEfSe was conducted to identify the main indicator species in bulk soil, rhizosphere soil, and roots across different treatments (**Figure 5**). The most diverse microbial community was found in bulk soil, followed by rhizosphere soil and roots. The bacterial genera *Dyella* (LDA = 4.52), *Massilia* (LDA = 4.23), *Acidibacter* (LDA = 3.40), *Acidovorax* (LDA = 3.29), and *Afipia* (LDA = 3.27) were abundantly present in bulk soil in C1 (**Figure 5A**). In bulk soil in SC1, *Burkholderia*–*Caballeronia*–*Paraburkholderia* (LDA = 3.92), *Paenibacillus* (LDA = 3.77), *Sporosarcina* (LDA = 3.68), *Bradyrhizobium* (LDA = 3.56), and *Solibacillus* (LDA = 3.36) were abundant. Bacillus (LDA = 4.54), *Nocardioides* (LDA = 3.68), *Pseudolabrys* (LDA = 3.46), *Bryobacter* (LDA = 3.33), and *Nitrosospira* (LDA = 3.30) were significantly enriched in bulk soil in SC2. Furthermore, the predominant bacterial genera in the rhizosphere soil from C1, SC1, and SC2 were *Dyella* (LDA = 4.46), *Burkholderia*–*Caballeronia*–*Paraburkholderia* (LDA = 4.60), and *Bacillus* (LDA = 4.83), respectively (**Figure 5B**). In roots, *Sphingomonas* (LDA = 4.70) was the most abundant in SC2, while *Burkholderia*–*Caballeronia*–*Paraburkholderia* (LDA = 5.16) were the most abundant in SC1. Furthermore, only a few microorganisms were significantly aggregated in roots in C1 (**Figure 5C**).

We examined variations in the distribution of bacterial species across bulk soil, rhizosphere soil, and roots within identical treatments utilising Venn diagrams (**Figures 5D–F**). In C1, *Dyella* was present in bulk soil, rhizosphere soil, and root samples. There were two genera common to rhizosphere soil and root samples, and four to bulk soil and rhizosphere soil in C1 (**Figure 5D**). In SC1, *Burkholderia*–*Caballeronia*–*Paraburkholderia* were common in bulk soil, rhizosphere soil, and root samples. In SC1, the number of common bacterial genera in bulk soil and rhizosphere soil increased to five (**Figure 5E**). In SC2, *Bacillus* was common in bulk soil, rhizosphere soil, and root samples, with increased abundance. In SC2, the total number of bacterial genera common to bulk soil and rhizosphere soil increased to seven: *Nocardioides*, *Pseudolabrys*, *Nitrosospira*, *Streptomyces*, *Actinomadura*, *Irregularibacter*, and *Cellulosimicrobium* (**Figure 5F**).

We identified 10, 11, and 13 fungal indicator species in bulk soil in C1, SC1, and SC2, respectively (**Supplementary Figure 3**). Fungal indicator species with high relative abundance included *Cladosporium* (LDA = 5.33) and *Ascitendus* (LDA = 4.10) in C1 bulk soil, *Pseudaleuria* (LDA = 4.47) and *Chordomyces* (LDA = 4.40) in SC1 bulk soil, and *Conocybe* (LDA = 4.01) and *Polyschema* (LDA = 3.98) in SC2 bulk soil (**Supplementary Figure 3A**). For rhizosphere soil, *Emericellopsis* (LDA = 4.13) and *Chaetomium* (LDA = 3.96) were highly abundant in SC2, while *Pseudaleuria* (LDA = 3.94) and *Melanconiella* (LDA = 3.32) were highly abundant in SC1 (**Supplementary Figure 3B**). Furthermore, *Atractiella* (LDA = 4.20) was enriched in the SC1 roots, while *Conocybe* (LDA = 3.69) and *Codinaea* (LDA = 3.29) were enriched in the SC2 roots (**Supplementary Figure 3C**). We conducted a further analysis of the distribution patterns of fungal species in bulk soil, rhizosphere soil, and root samples within the same treatments utilising Venn diagrams (**Supplementary Figures 3D–F**). Compared with C1 and SC1 groups, the SC2 group exhibited the presence of shared fungal genera across bulk soil, rhizosphere soil, and root samples and shared fungal genera between bulk soil and rhizosphere soil, between rhizosphere soil and roots. This indicates that after two years of rotation between *C. yanhusuo* and soybean, a more complex and stable interaction network has formed among the microbial communities.

### 3.6 Effects of soybean–*C. yanhusuo* rotation on soil microbial co-occurrence networks

**Figure 6:**
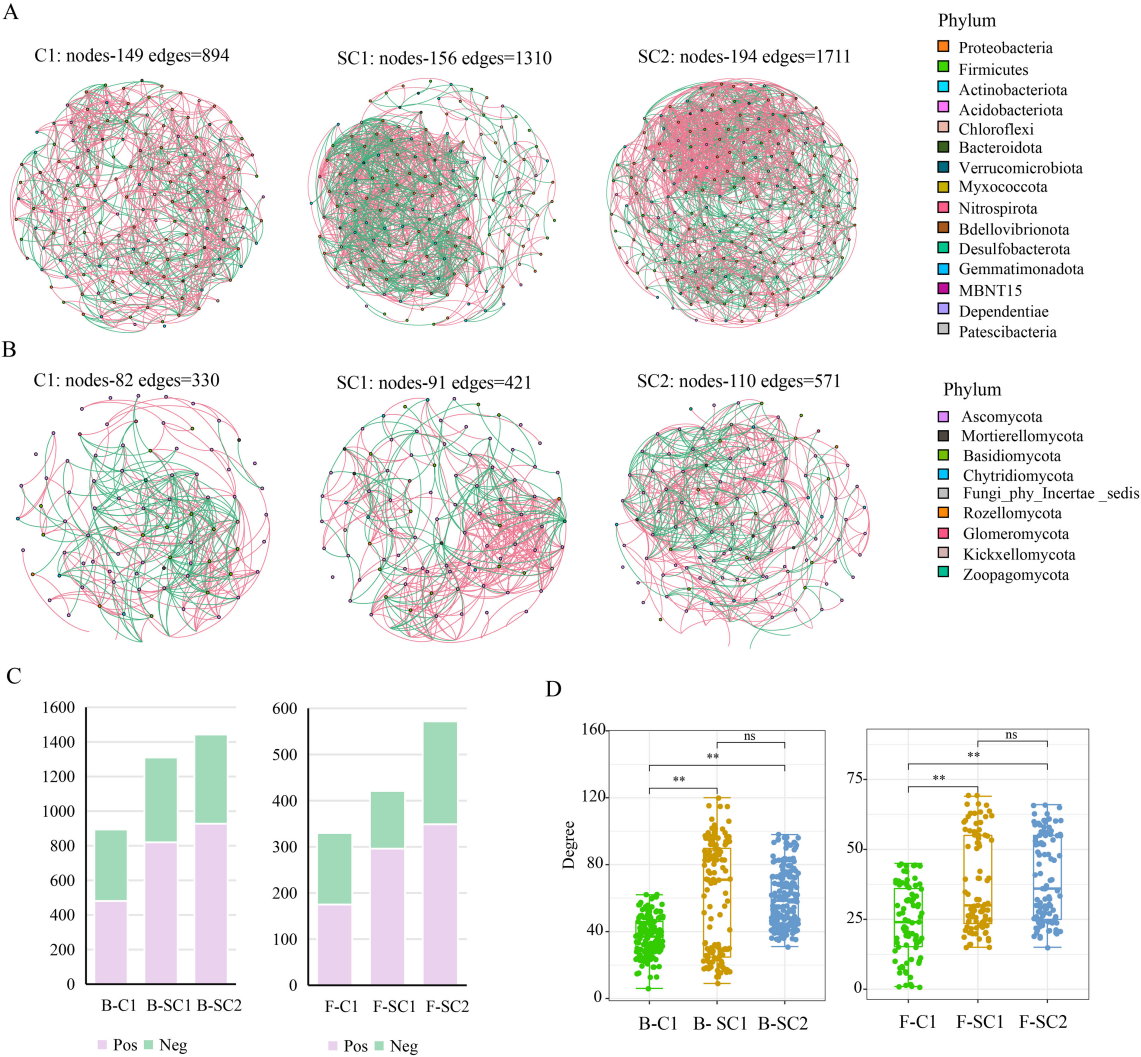
Co-occurrence network analysis of soybean–*C. yanhusuo* rotation soil microbial communities. **(A)** Bacteria, **(B)** Fungi. Nodes represent identified bacterial and fungal genera; the size of each node correlates with the number of connections, while the colour denotes the corresponding phylum. Spearman, |r| > 0.8, *P* < 0.05. **(C)** Proportion of positively or negatively linked edges inside the microbial co-occurrence network. **(D)** Degrees of freedom of network nodes. B, Bacteria; F, Fungi.

We constructed co-occurrence networks of bacteria and fungi to assess the effects of soybean–*C. yanhusuo* rotation on bacterial and fungal communities (**Figure 6**). Compared with C1, SC1 and SC2 increased the complexity and density of the microbial network.

With the extension of crop rotation period, the complexity and density of microbial networks exhibited a gradual increase. The number of bacteria identified in the networks increased from 149 in C1 to 156 in SC1 and to 194 in SC2, while that of fungi increased from 82 in C1 to 91 in SC1 and to 110 in SC2. Network complexity in the bacterial network increased from 894 in C1 to 1,711 in SC2, and the complexity of the fungal network increased from 330 in C1 to 571 in SC2 (**Figures 6A, B**). The average path length of the bacterial network decreased from 3.34 in C1 to 2.84 in SC2, while the length of the fungal network decreased by 11.05% from C1 to SC2 (**Supplementary Table 47**). The modularity of the bacterial network increased from 0.44 in C1 to 0.53 in SC2, and the modularity of the fungal network increased by 4.44% from C1 to SC2 (**Supplementary Table 47**). The number of positive links between the bacterial community networks increased in both SC1 and SC2, with increases from 53.03% in C1 to 61.01% in SC2. The number of positive links between fungal community networks increased from 53.80% in C1 to 64.24% in SC2 (**Figure 6C**).

The degrees of freedom of the network nodes in the fungal and bacterial networks also increased in SC1 and SC2 (**Figure 6D**). The findings indicate that the coexistence networks of fungal and bacterial communities are markedly affected by prior crops and production practices.

### 3.7 Functions of bacterial and fungal communities under soybean–*C. yanhusuo* rotation

**Figure 7:**
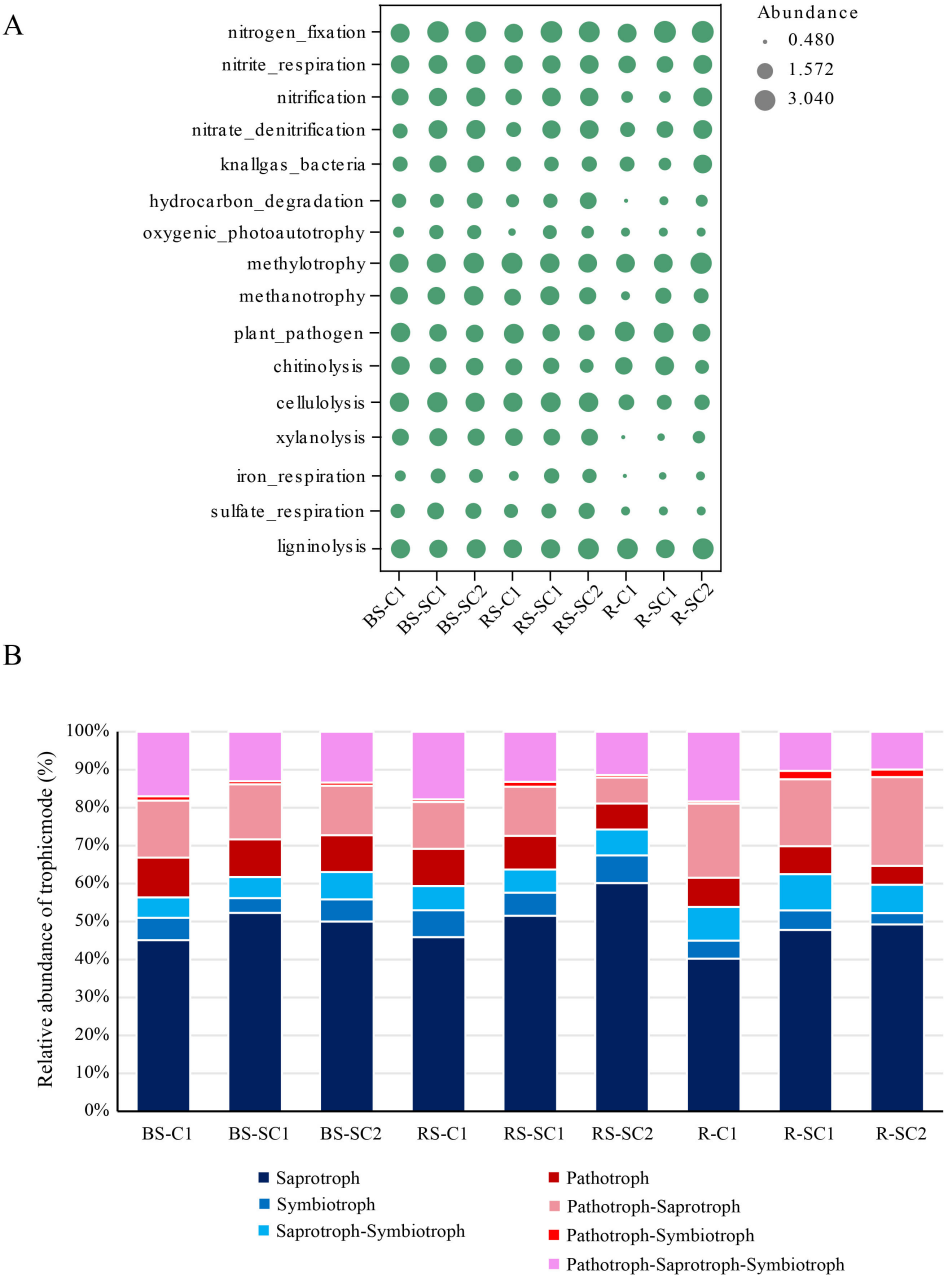
Functional shift of bacterial and fungal communities in response to different cropping practices. Variations in key ecological function profiles of bacterial communities **(A)** and fungi communities **(B)**.

The ecological roles of microbial communities were forecasted utilising FAPROTAX and FUNGuild. The functional characteristics of microbial communities differed significantly between the different treatments. Bacterial taxa were mainly associated with nitrate denitrification, hydrocarbon degradation, and plant pathogens (**Figure 7A**). Regardless of whether it is bulk soil, rhizosphere soil, or root samples, compared with C1, bacterial groups responsible for nitrate denitrification and hydrocarbon degradation significantly increased in SC1 and SC2, with those in SC2 being more abundant than those in SC1. In the bulk soil, rhizosphere soil, and root samples of SC2, there was a notably high functional allocation for cellulolysis, xylanolysis, iron_respiration, and sulfate_respiration. Notably, the abundance of bacterial groups associated with plant pathogens was reduced in SC2 relative to that in C1.

Fungal taxa were divided into several major groups: saprotrophs, symbiotrophs, saprotroph–symbiotrophs, pathotrophs, pathotroph–saprotrophs, pathotroph–symbiotrophs, and pathotroph–saprotroph–symbiotrophs (**Figure 7B**). In bulk soil, rhizosphere soil, and root samples, the fungal communities were predominantly composed of saprotrophs, followed by pathotroph–saprotrophs. All fungal functional characteristics related to pathotrophs significantly decreased in SC1 and SC2. Among them, the fungal functional characteristics of pathotroph–saprotroph–symbiotrophs exhibited the most pronounced decrease across bulk soil, rhizosphere soil, and root samples in SC2.

### 3.8 Relationship between rhizosphere microbial communities and soil chemical properties, yield, and active components of *C. yanhusuo*

**Figure 8:**
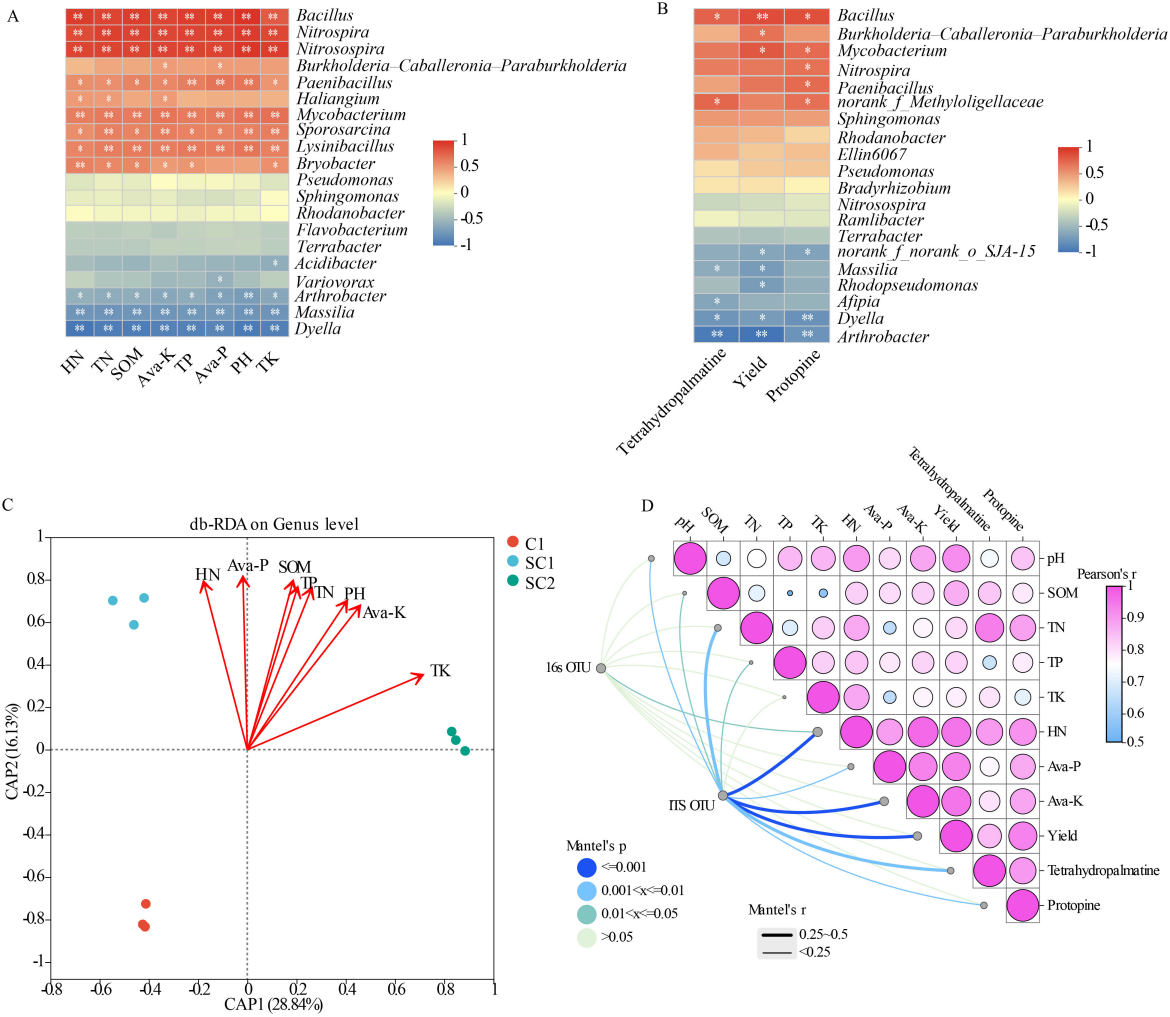
Effect of different rotation years on *C*. *yanhusuo* and relationship between microorganisms, soil factors, yield and active ingredients of *C*. *yanhusuo*. Correlation heatmap of bacterial genera with soil factors **(A)** and with active ingredients and yield of *C*. *yanhusuo***(B)**. **(C)** Distance-based redundancy analysis on soil physicochemical properties and soil microbial communities of *C*. *yanhusuo* in Bacteria. **(D)** Mantel test between soil properties, active ingredients, and microbial diversity. *P < 0.05; **P < 0.01.

We analysed correlations among dominant microbial genera, soil parameters, and yield, as well as active ingredients, using Pearson’s correlation analysis. The bacterial genera *Bacillus*, *Nitrospira*, *Nitrosospira*, *Paenibacillus*, *Mycobacterium*, *Sporosarcina*, and *Lysinibacillus* positively correlated with various soil parameters. *Bacillus* also positively correlated with yield and the contents of tetrahydropalmatine and protopine. *Mycobacterium* positively correlated with yield and protopine, and *Nitrospira* and *Paenibacillus* also positively correlated with protopine. *Arthrobacter*, *Massilia*, and *Dyella* negatively correlated with soil parameters, and *Arthrobacter* and *Dyella* negatively correlated with yield and the contents of tetrahydropalmatine and protopine (**Figures 8A, B**). The fungal genera *Mortierella*, *Trichoderma*, and *Podospora* positively correlated with soil parameters, and with yield and the contents of tetrahydropalmatine and protopine. *Chaetomium* positively correlated with soil parameters, and with the contents of tetrahydropalmatine and protopine (**Supplementary Figures 4A, B**). *Alternaria*, *Myrmecridium*, *Epicoccum*, and *Cladosporium* negatively correlated with soil parameters, and with yield and the contents of tetrahydropalmatine and protopine (**Supplementary Figures 4A, B**).

Distance-based redundancy analysis was employed to investigate correlations between soil physicochemical parameters and inter-sample variations in the different experimental treatments (**Figure 8C**, **Supplementary Figure 5**). SC1 showed a strong positive correlation with HN, SOM, and Ava-P, while SC2 showed a strong positive correlation with soil pH, TN and Ava-K. In contrast, C1 displayed negative correlations with many soil parameters. Mantel test analysis revealed environmental factors influencing the assembly of bacterial and fungal communities. The richness of soil bacterial OTUs was strongly correlated with HN (**Figure 8D**), while fungal OTUs were positively correlated with soil HN and Ava-K, as well as with the yield of *C. yanhusuo* and the tetrahydropalmatine content.

**Figure 9:**
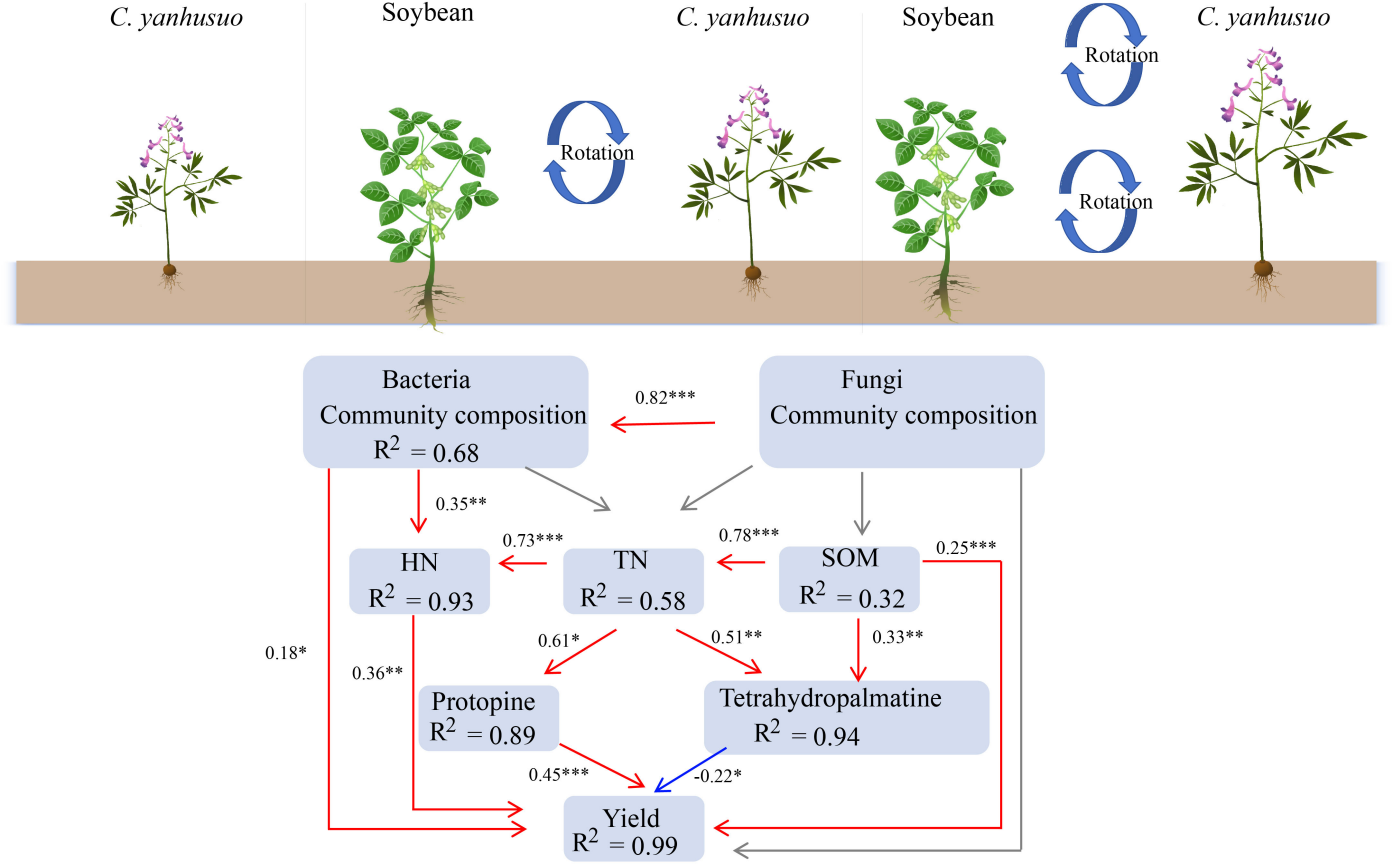
Structural equation model (SEM) showing the direct and indirect effects of crop rotation on soil nutrients, microbial communities, and the yield and quality of *C. yanhusuo*. Microbial community composition was represented using the PCoA profiles. The red arrows signify notable positive correlations, the blue arrows signify notable negative correlations, while the grey arrows denote pathways that are not statistically significant. The models achieved acceptable fit with AIC = 270.21 and Fish’s C = 0.46. *P < 0.05, **P < 0.01; ***P < 0.001.

SEM was employed to evaluate the impact of crop rotation on soil nutrients, microbial populations, as well as the subsequent crop yield and quality; this model accurately represented the data (**Figure 9**; **Supplementary Tables 48**, **49**). The model indicated that crop rotation influenced soil nutrients and the yield and quality of subsequent crops by affecting microbial communities. The composition of fungal communities was directly positively correlated with that of bacterial communities. The composition of bacterial communities was directly positively correlated with yield and HN, and HN was directly positively correlated with yield. SOM was positively correlated with yield, the tetrahydropalmatine content, and TN. TN in turn was positively correlated with tetrahydropalmatine and protopine, and showed a direct positive correlation with HN. Crop rotation exerted a positive influence on the yield of subsequent crops.

## 4 Discussion

### 4.1 Soybean–*C. yanhusuo* rotation synergistically enhances quality and yield of *C. yanhusuo*, as well as soil nutrient availability and enzyme activities

The soybean–*C. yanhusuo* rotation system demonstrates a dual capacity to enhance medicinal crop performance. Compared to C1, crop rotation (SC1, SC2) significantly increased the yield and active medicinal components in the tubers of *C. yanhusuo*. In SC2, the yield and contents of tetrahydropalmatine and protopine increased by 56.04%, 26.69%, and 40.97%, respectively, compared to those in C1 (**Figure 2**). These results highlight that crop rotation improves the quality of *C. yanhusuo* without diminishing yield by optimising the growth environment of the crops. The parallel improvement in alkaloid content aligns with studies linking soil health to secondary metabolite synthesis in medicinal plantsement (Fierer, 2017; Hartman et al., 2018).

Soybean rotation alleviates the reduction and imbalance in soil nutrients from continuous monocropping (Luo et al., 2024). Soybean–*C. yanhusuo* rotation strongly enhanced soil nitrogen and nutrient availability through nitrogen fixation and improved soil biological activity. With an increasing number of years of soybean–*C. yanhusuo* rotation, the soil TN and HN contents gradually increased. The HN content in SC2 was substantially higher than that in C1 and SC1. The TN content was the highest in SC2. However, no significant differences were observed among SC1, C1, and the initial state before treatment. The nitrogen-fixing ability of soybean is a key reason for this. Leguminous crops form a symbiotic relationship with rhizobia, which can fix atmospheric nitrogen and convert it into forms usable by plants (Yeremko et al., 2025). With an increasing number of rotation years, fixed nitrogen gradually accumulates and increases the soil nitrogen content (Yan et al., 2024). However, the lack of significant differences among SC1 and C1 may be because TN is relatively stable. Significant changes in TN require longer timescales and relatively high nitrogen inputs or outputs.

Crop rotation may promote nutrient availability by increasing soil biological activity (Wang et al., 2023). Some substances secreted by soybean roots may stimulate the activity of soil microorganisms, accelerating nutrient release (Yu et al., 2024). Compared with C1, SC1 and SC2 had higher activities of S-SC, S-UE, and S-ACP, with the highest enzyme activities observed in SC2. Soil enzymes are catalysts of soil biochemical processes, and their activities reflect the intensity of soil material transformation and energy flow (Schnecker et al., 2014; Yang et al., 2020). Diverse crop rotations may change the biomass and diversity of soil microorganisms, affecting enzyme secretion and activity. Soybean root rhizodeposits may provide rich carbon sources and energy for soil microorganisms, promoting their growth and reproduction and enhancing enzyme activities. From rotational years 1 to 2, this promoting effect became more pronounced, and the highest enzyme activities were observed in SC2.

Different crops selectively absorb and use nutrients. Crop rotation can inhibit excessive absorption of a certain nutrient by a single crop, which maintains the balance and supply of soil nutrients (Malik et al., 2025). The Ava-P, and Ava-K contents were the highest in SC2, followed by SC1. Before treatment, the contents of these parameters were slightly, but not significantly, higher than those in C1. The slightly higher Ava-P, and Ava-K contents before treatment relative to C1 may be attributed to the unbalanced consumption of soil nutrients during monocropping. This results in a relatively poor soil nutrient status. Monocropping in C1 leads to continuous and large-scale absorption of soil nutrients. Over time, this imbalance leads to a decline in soil fertility and crop yield reduction. Alternating cultivation of different crops changes nutrient absorption patterns. Therefore, crop rotation enables a more efficient and sustainable use of soil nutrients and inhibits excessive consumption of a certain nutrient by a single crop (Al-Musawi et al., 2025). Meanwhile, crop rotation may also promote the cycling and regeneration of soil nutrients.

There is a complex relationship between crop rotation and soil nutrient content. Higher crop yields would lead to increased nutrient uptake, reducing the soil nutrient content. However, after soybean rotation, while increasing crop yields, it could also enhance the content of SOM and other nutrients in the soil. This might be because, with the increasing number of rotation years, the accumulation of fixed nitrogen gradually increases. This increases the soil nitrogen content, which provides favourable conditions for SOM synthesis. The root systems of soybean and *C. yanhusuo* secrete a variety of organic substances. These secretions can stimulate soil microorganism activity, promoting their decomposition and transformation of soil organic residues. After the decomposition of soybean and *C. yanhusuo* soil residues, a large quantity of organic substances is released. These organic substances increase the SOM content and improve the soil chemical properties.

The study found that soil bulk density and porosity remained largely unchanged across different rotation years, indicating that crop rotation had limited impact on soil physical properties. Soil bulk density and porosity did not significant differ between C1, SC1, and SC2, or the initial state before treatment. Soil bulk density in C1 increased slightly, but not significantly, compared with that before treatment. Soil physical properties are influenced by various factors, including soil texture, organic matter content, and tillage methods (Simon et al., 2025). Although crop rotation had a certain impact on soil biological and chemical properties in this study, it did not significantly influence the physical structure of the soil. The slight increase in soil bulk density in C1 may have been due to soil compaction caused by monocropping. However, owing to the limited time and spatial scales covered in this study, this change was not significant.

### 4.2 Soybean–*C. yanhusuo* rotation reconstructs microbial diversity and community structure

The rhizosphere microbial community is crucial for plant development and ecosystem functionality (Schmidt et al., 2019; Tian et al., 2020; Zeeshan Ul Haq et al., 2023). We evaluated the impact of soybean–*C. yanhusuo* rotation on microbial α-diversity and community composition in bulk soil, rhizosphere soil, and root. Prolonged rotation periods significantly enhanced microbial diversity and restructured community assembly, with the SC2 group exhibiting the most pronounced effects.

Microbial α-diversity, quantified by Chao-1 and Shannon indices, was considerably greater in rotation treatments than monocropping. In bulk soil, bacterial richness in SC2 increased by 13.31% relative to C1, while fungal richness rose by 34.08%. Similarly, rhizosphere soil and roots in SC1 and SC2 exhibited elevated bacterial and fungal diversity compared to those in C1 (**Figure 3**). These findings align with those of prior studies. For example, a study on wheat-pea rotations reported an increase in bacterial richness after two years of rotation (Woo et al., 2022), while our study observed a 13.31% increase in bacterial richness in bulk soil after a two-year soybean–*C. yanhusuo* rotation. The greater magnitude of fungal diversity enhancement (34.08% in SC2) is particularly noteworthy, as fungal communities are often more sensitive to crop rotation than bacterial communities. This sensitivity may stem from fungi’s reliance on stable carbon sources, which are better provided by diverse rotation systems (Zong et al., 2024).

Bulk soil, rhizosphere soil, and root samples separated along the first principal component axis, reflecting niche-specific microbial assembly. SC2 and C1 were the farthest apart on the PCoA map, indicating that prolonged rotation drives significant divergence in microbial community structure (**Figure 3**). However, our study extends these findings to medicinal plant systems, demonstrating that rotation can induce profound shifts. The structure and dynamics of microbial communities in different samples are essential markers of ecosystem performance and plant health (Fracchia et al., 2024). Soybean–*C. yanhusuo* rotation significantly reshaped microbial community structures (**Figure 4**). In bulk and rhizosphere soil, the bacterial phyla Proteobacteria and Firmicutes exhibited opposing trends under rotation. While Proteobacteria dominated in C1, their relative abundance declined with rotation duration, accompanied by a marked increase in Firmicutes. This shift aligns with prior findings on legume rotations (Yu et al., 2021). Furthermore, our results suggest that short-term rotation temporarily disrupts fungal equilibrium, but prolonged rotation restores balance. Notably, *Trichoderma* increased in rhizosphere soil, correlating with reduced pathogen loads, and so did beneficial taxa, such as *Mortierella*.

The indicator species analysis revealed distinct microbial assembly patterns across soil compartments and rotation durations, highlighting the ecological restructuring induced by soybean–*C. yanhusuo* rotation (**Figure 5**). In bulk soil, the transition from C1 to rotation systems (SC1, SC2) drove a shift from pathogenic to beneficial bacterial genera. C1 bulk soil was dominated by *Dyella* and *Massilia*, taxa often associated with nutrient-poor soils and mild plant pathogenesis. Conversely, SC1 and SC2 bulk soil showed enrichment of *Burkholderia*–*Caballeronia*–*Paraburkholderia* and *Bacillus*, known for nitrogen fixation and biocontrol activities (Caulier et al., 2019). Notably, *Bradyrhizobium* in SC1 bulk soil, a classic legume symbiont, underscores soybean’s role in enhancing nitrogen availability (Qiao et al., 2024).

In C1 bulk soil, *Cladosporium* and *Ascitendus*, potential plant pathogens, dominated. SC1 and SC2 bulk soil shifted to *Conocybe*, saprotrophic fungi critical for organic matter decomposition (Asif et al., 2025). Rhizosphere soil in SC2 was enriched in *Emericellopsis* and *Chaetomium*, known for their biocontrol and lignin-degrading capabilities (Agrawal and Saha, 2022; Tian and Li, 2022). Root fungal communities in SC2 exhibited *Conocybe* and *Codinaea*, suggesting enhanced organic matter recycling (Réblová et al., 2021). In conclusion, soybean–*C. yanhusuo* rotation restructures microbial communities and fosters functional redundancy and pathogen resistance, positioning it as a sustainable strategy for high-value crop cultivation.

Through the construction of a co-occurrence network for soil bacteria and fungus (**Figure 6**) and the use of FAPROTAX and FUNGILD functional analyses (**Figure 7**), we determined that crop rotation reconfigured the topological structure of the microbial network and substantially modified the functions of the microbial community. Bacterial communities under rotation exhibited a marked expansion of functional traits linked to nutrient cycling. In both SC1 and SC2, the abundance of taxa responsible for nitrate denitrification and hydrocarbon degradation increased progressively, aligning with elevated SOM and nitrogen availability in rotation systems. SC2 soils showed heightened functional allocation to cellulolysis, xylanolysis, iron respiration, and sulphate respiration, indicating a diversification of organic matter decomposition pathways. Fungal communities underwent a functional transition from pathogenic to saprotrophic dominance. Across all compartments, saprotrophs remained the dominant guild, but rotation dramatically reduced pathogen-related groups, with SC2 showing the steepest decline. This suppression of pathogenic fungi (e.g., *Fusarium*) aligns with observed increases in biocontrol taxa. This may create an ecological barrier against pathogenic microorganisms by strengthening the relationships among beneficial microorganisms.

### 4.3 Association between microbial communities and soil indicators under crop rotation

In this study, distance-based redundancy analysis was employed in the different experimental treatments (**Figure 8**). SC1 was strongly positively correlated with HN, SOM, and Ava-P. This indicates that these soil nutrients were closely associated with the 1-year soybean–*C. yanhusuo* rotation. Crop rotation might have promoted the accumulation of these nutrients or maintained their contents. SC2 was strongly positively correlated with soil pH, TN, and Ava-K. By comparison, C1 was negatively correlated with many soil parameters. This highlights that monocropping has adverse effects on soil physicochemical properties. Mantel test analysis revealed that the richness of soil bacterial OTUs was strongly correlated with HN. Fungal OTUs were positively correlated with soil HN, Ava-K, tetrahydropalmatine, and *C. yanhusuo* yield. This indicates that fungal communities were not only influenced by soil nutrients but also closely related to crop yield and quality. Fungi play a crucial role in soil nutrient cycling and crop growth responses. Further analysis revealed that soil HN, Ava-P, Ava-K, and SOM were positively correlated with *C. yanhusuo* yield, providing a theoretical basis for improving crop yield by regulating soil nutrients. The increase in crop growth requires an increase in nutrient absorption, which may reduce the soil nutrient content. This then leads to a negative correlation between yield and soil nutrient content. The contents of TN, SOM, HN, Ava-K and Ava-P in the soil are positively correlated with the yield. This phenomenon might be related to the overall improvement of the soil system during crop rotation. Crop rotation not only affected crop growth but also had a positive impact on soil microbial communities. Soil microorganisms play a key role in nutrient cycling and transformation processes (Pradhan et al., 2025). They can decompose organic matter and release nutrients for crop absorption and utilisation. Under crop rotation, the types and quantities of soil microorganisms might have changed, and their activity increased, thereby promoting the transformation and availability of soil nutrients (Cui et al., 2024).

Studies have shown that the biomass of soil organisms and plants plays a crucial role in the weathering of soil minerals (Sokolova, 2011). Microorganisms, plant roots, and soil fauna can secrete various organic acids and enzymes. These substances can dissolve soil minerals and release the nutrients. Organic acids such as oxalic acid and citric acid secreted by microorganisms can react chemically with soil minerals and release elements such as phosphorus and potassium and convert them into forms usable by plants (Menezes-Blackburn et al., 2016). Plant roots secrete root exudates during growth that promote the weathering of soil minerals (Chen et al., 2022). In crop rotation systems, the biomass of plants and soil organisms may be higher and more diverse. Interactions among these different organisms enhance the weathering of soil minerals, thereby increasing the availability of soil nutrients and providing nutrient sources for crop growth. The soybean–*C. yanhusuo* rotation creates a virtuous cycle in which improved soil health promotes the development of beneficial microbial communities, which in turn enhances crop yield and quality.

SEM were utilised to assess the impact of crop rotation on soil nutrients, microbial populations, as well as the yield and quality of subsequent crops (**Figure 9**). The model indicates that crop rotation influenced soil nutrients and the yield and quality of subsequent crops by affecting microbial communities. The composition of fungal and bacterial communities was directly and positively correlated, suggesting a close interaction and synergistic relationship between soil fungi and bacteria. They jointly participate in nutrient cycling and transformation, mutually promoting growth and reproduction. The composition of bacterial communities was directly positively correlated with yield and HN. Bacteria can decompose organic matter and release nutrients such as nitrogen, thereby providing nutrition for crops and increasing crop yields. The positive correlation between HN and yield further confirms the importance of nitrogen for crop growth. SOM was positively correlated with yield, tetrahydropalmatine, and TN, indicating that SOM is not only an important nutrient source for crop growth but also has a positive impact on crop quality. TN was positively correlated with tetrahydropalmatine, protopine, and HN. This suggests a close connection between TN and HN in soil, and that nitrogen has a significant impact on both the yield and quality of crops. Crop rotation had a positive effect on the yield of subsequent crops, providing strong evidence for the promotion of crop rotation systems in practical agricultural production.

To date, research has primarily centred on the changes in major soil nutrients, with relatively limited attention given to the micronutrient dynamics. Although micronutrients account for a relatively small proportion in the soil and plant growth, they have a non-negligible impact on the physiological functions, growth and development, and the final yield and quality of plants. We plan to prioritise measuring micronutrients in future research. Through more in-depth analysis, we aim to further uncover the influence mechanism of crop rotation on soil micronutrients.

## 5 Conclusions

This study examined the regulating systems underlying the soybean–*C. yanhusuo* rotation system. Yield and active component content of *C. yanhusuo* improved with rotation duration. Meanwhile, this rotation significantly increased TN, HN, and SOM contents. In the soybean–*C. yanhusuo* rotation system, the abundance of Proteobacteria decreased with the increase of rotation years, while the abundance of Firmicutes significantly increased. Functional predictions indicated enhanced functions related to nitrate denitrification, cellulolysis and xylanolysis compared to monoculture systems, while plant pathogen-related functional groups decreased. Pearson correlation analysis revealed significant positive correlations between dominant genera, such as *Bacillus*, and active ingredients, as well as soil nutrient indicators. SEM revealed that the composition of microbial communities promoted increases in the yield of subsequent crops. Moreover, the composition of microbial communities affected HN, which further enhanced yield. Additionally, SOM and TN were related with an improved quality of subsequent crops. Compared with monoculture, the rotation of soybean–*C. yanhusuo* improved yield and active component contents and soil nutrients, optimised the structure of soil microbial communities, and provided a new perspective for the increase in yield and quality of crop rotation.

## Data Availability

The original contributions presented in the study are publicly available. This data can be found here: NCBI Short Read Archive (SRA), SRR33853140 to SRR33853193.
