# Supplementary material for: Rotation of soybean and Corydalis yanhusuo enhances yield and active compounds of C. yanhusuo via soil nutrient optimisation and rhizosphere microbiome engineering
Source: Front Plant Sci. 2025 Dec 9;16:1692138. doi: 10.3389/fpls.2025.1692138 (PMC12722525; doi:10.3389/fpls.2025.1692138)
Supplement: Supplementary file 1 [file Supplementaryfile1.docx]

Supplementary Material

# Supplementary Tables and Figures

**Supplementary Table 1. Soil physical properties before and after treatment.**

| Treatments | Soil Bulk Density(g/cm3) | Soil Porosity(%) |
| --- | --- | --- |
| BE-1 | 1.160 | 56.230 |
| BE-2 | 1.180 | 55.470 |
| BE-3 | 1.200 | 54.720 |
| C1-1 | 1.200 | 54.720 |
| C1-2 | 1.230 | 53.580 |
| C1-3 | 1.210 | 54.340 |
| SC1-1 | 1.160 | 56.230 |
| SC1-2 | 1.150 | 56.600 |
| SC1-3 | 1.190 | 55.090 |
| SC2-1 | 1.190 | 55.090 |
| SC2-2 | 1.140 | 56.980 |
| SC2-3 | 1.150 | 56.600 |

**Note:** BE, Before experiment;C1, monoculture of *C. yanhusuo*; SC1, soybean and *C. yanhusuo* rotation for one year; SC2, soybean and *C. yanhusuo* rotation for two years.

**Supplementary Table 2.** Soil chemical properties before and after treatment.

| Treatments | pH | SOM(g/kg) | TN(g/kg) | TP(g/kg) | TK(g/kg) | HN(mg/kg) | Ava-P(mg/kg) | Ava-K(mg/kg) |
| --- | --- | --- | --- | --- | --- | --- | --- | --- |
| BE-1 | 6.45 | 12.96 | 1.668 | 0.651 | 7.267 | 212.80 | 48.34 | 366.67 |
| BE-2 | 6.55 | 11.45 | 1.663 | 0.653 | 7.300 | 211.40 | 43.11 | 360.00 |
| BE-3 | 6.50 | 13.24 | 1.646 | 0.611 | 7.433 | 207.40 | 44.66 | 370.00 |
| C1-1 | 5.30 | 11.84 | 1.688 | 0.652 | 7.100 | 196.00 | 43.03 | 358.33 |
| C1-2 | 5.70 | 11.37 | 1.663 | 0.653 | 7.133 | 197.40 | 45.03 | 360.00 |
| C1-3 | 6.00 | 13.31 | 1.688 | 0.634 | 7.167 | 194.60 | 41.73 | 351.67 |
| SC1-1 | 6.20 | 16.30 | 1.680 | 0.663 | 7.233 | 222.60 | 50.17 | 390.83 |
| SC1-2 | 6.70 | 14.32 | 1.690 | 0.677 | 7.167 | 221.20 | 51.72 | 393.33 |
| SC1-3 | 6.90 | 13.08 | 1.700 | 0.720 | 7.433 | 225.40 | 50.03 | 385.00 |
| SC2-1 | 7.30 | 15.83 | 1.758 | 0.720 | 7.370 | 247.80 | 51.15 | 411.67 |
| SC2-2 | 6.80 | 17.54 | 1.752 | 0.685 | 7.447 | 242.20 | 54.88 | 395.66 |
| SC2-3 | 7.00 | 15.95 | 1.764 | 0.692 | 7.256 | 250.60 | 52.01 | 380.00 |

Note: Ava-P, available phosphorus; Ava-K, available potassium; HN, hydrolysable nitrogen; SOM, soil organic matter; TN, total nitrogen; TP, total phosphorous; TK, total potassium

**Supplementary Table 3. Shapiro-Wilk test results for soil physical properties before and after treatment.**

| Variable | Test Method | Statistic | P-value |
| --- | --- | --- | --- |
| Soil Bulk Density(g/cm3) | Shapiro-Wilk | 0.950 | 0.635 |
| Soil Porosity(%) | Shapiro-Wilk | 0.949 | 0.629 |

Note: *P*-values < 0.05 indicate deviation from normality.

**Supplementary Table 4. Levene’s test results for variance homogeneity across soil physical properties before and after treatment.**

| Variable | Test Method | Statistic | P-value |
| --- | --- | --- | --- |
| Soil Bulk Density(g/cm3) | Levene’s | 0.491 | 0.698 |
| Soil Porosity(%) | Levene’s | 0.487 | 0.701 |

Note: *P*-values < 0.05 indicates significant heterogeneity of variance.

**Supplementary Table 5. One-Way ANOVA results for soil bulk density across treatment groups**

| Source | SS | df | MS | F | P-value |
| --- | --- | --- | --- | --- | --- |
| Between Treatments | 0.005 | 3 | 0.002 | 3.824 | 0.057 |
| Within Treatments | 0.004 | 8 | 0 |  |  |
| Total | 0.009 | 11 |  |  |  |

Note: *P*-values < 0.05 indicates significant differences among groups.

**Supplementary Table 6. Post-Hoc comparisons of soil bulk density among treatment groups (Tukey HSD Test).**

| Comparison | Mean Difference | P-value |
| --- | --- | --- |
| BE vs. C1 | -0.033 | 0.284 |
| BE vs. SC1 | 0.013 | 0.863 |
| BE vs. SC2 | 0.020 | 0.663 |
| C1 vs. SC1 | 0.046 | 0.099 |
| C1 vs. SC2 | 0.053 | 0.057 |
| SC1 vs. SC2 | 0.007 | 0.979 |

Note: *P*-values < 0.05 indicates significant differences.

**Supplementary Table 7. One-Way ANOVA results for soil porosity across treatment groups**

| Source | SS | df | MS | F | P-value |
| --- | --- | --- | --- | --- | --- |
| Between Treatments | 7.200 | 3 | 2.400 | 3.801 | 0.058 |
| Within Treatments | 5.052 | 8 | 0.631 |  |  |
| Total | 12.252 | 11 |  |  |  |

Note: *P*-values < 0.05 indicates significant differences among groups.

**Supplementary Table 8. Post-Hoc comparisons of soil porosity among treatment groups (Tukey HSD Test).**

| Comparison | Mean Difference | P-value |
| --- | --- | --- |
| BE vs. C1 | 1.26 | 0.284 |
| BE vs. SC1 | -0.5 | 0.866 |
| BE vs. SC2 | -0.75 | 0.668 |
| C1 vs. SC1 | -1.76 | 0.100 |
| C1 vs. SC2 | -2.01 | 0.058 |
| SC1 vs. SC2 | -0.25 | 0.979 |

Note: *P*-values < 0.05 indicates significant differences.

**Supplementary Table 9. Shapiro-Wilk test results for soil chemical properties before and after treatment.**

| Variable | Test Method | Statistic | P-value |
| --- | --- | --- | --- |
| pH | Shapiro-Wilk | 0.966 | 0.863 |
| SOM(g/kg) | Shapiro-Wilk | 0.924 | 0.325 |
| TN(g/kg) | Shapiro-Wilk | 0.866 | 0.058 |
| TP(g/kg) | Shapiro-Wilk | 0.948 | 0.605 |
| TK(g/kg) | Shapiro-Wilk | 0.923 | 0.313 |
| HN(mg/kg) | Shapiro-Wilk | 0.921 | 0.295 |
| Ava-P(mg/kg) | Shapiro-Wilk | 0.928 | 0.362 |
| Ava-K(mg/kg) | Shapiro-Wilk | 0.945 | 0.570 |

Note: *P*-values < 0.05 indicate deviation from normality.

**Supplementary Table 10. Levene’s test results for variance homogeneity across soil physical properties before and after treatment.**

| Variable | Test Method | Statistic | P-value |
| --- | --- | --- | --- |
| pH | Levene’s | 1.798 | 0.225 |
| SOM(g/kg) | Levene’s | 0.505 | 0.690 |
| TN(g/kg) | Levene’s | 1.229 | 0.361 |
| TP(g/kg) | Levene’s | 1.615 | 0.261 |
| TK(g/kg) | Levene’s | 1.917 | 0.205 |
| HN(mg/kg) | Levene’s | 1.529 | 0.28 |
| Ava-P(mg/kg) | Levene’s | 1.235 | 0.359 |
| Ava-K(mg/kg) | Levene’s | 1.664 | 0.251 |

Note: *P*-values < 0.05 indicates significant heterogeneity of variance.

**Supplementary Table 11. One-Way ANOVA results for soil pH across treatment groups**

| Source | SS | df | MS | F | P-value |
| --- | --- | --- | --- | --- | --- |
| Between Treatments | 2.937 | 3 | 0.979 | 12.268 | 0.002 |
| Within Treatments | 0.638 | 8 | 0.080 |  |  |
| Total | 3.575 | 11 |  |  |  |

Note: *P*-values < 0.05 indicates significant differences among groups.

**Supplementary Table 12. Post-Hoc comparisons of soil pH among treatment groups (Tukey HSD Test).**

| Comparison | Mean Difference | P-value |
| --- | --- | --- |
| BE vs. C1 | 0.833 | 0.028 |
| BE vs. SC1 | -0.100 | 0.971 |
| BE vs. SC2 | -0.533 | 0.174 |
| C1 vs. SC1 | -0.933 | 0.016 |
| C1 vs. SC2 | -1.367 | 0.002 |
| SC1 vs. SC2 | -0.433 | 0.308 |

Note: *P*-values < 0.05 indicates significant differences.

**Supplementary Table 13. One-Way ANOVA results for SOM across treatment groups**

| Source | SS | df | MS | F | P-value |
| --- | --- | --- | --- | --- | --- |
| Between Treatments | 35.087 | 3 | 11.696 | 8.506 | 0.007 |
| Within Treatments | 11.000 | 8 | 1.375 |  |  |
| Total | 46.087 | 11 |  |  |  |

Note: *P*-values < 0.05 indicates significant differences among groups.

**Supplementary Table 14. Post-Hoc comparisons of SOM among treatment groups (Tukey HSD Test).**

| Comparison | Mean Difference | P-value |
| --- | --- | --- |
| BE vs. C1 | 0.377 | 0.978 |
| BE vs. SC1 | -2.017 | 0.230 |
| BE vs. SC2 | -3.890 | 0.015 |
| C1 vs. SC1 | -2.393 | 0.134 |
| C1 vs. SC2 | -4.267 | 0.009 |
| SC1 vs. SC2 | -1.873 | 0.279 |

Note: *P*-values < 0.05 indicates significant differences.

**Supplementary Table 15. One-Way ANOVA results for TN across treatment groups**

| Source | SS | df | MS | F | P-value |
| --- | --- | --- | --- | --- | --- |
| Between Treatments | 0.017 | 3 | 0.006 | 46.207 | 0 |
| Within Treatments | 0.001 | 8 | 0 |  |  |
| Total | 0.017 | 11 |  |  |  |

Note: *P*-values < 0.05 indicates significant differences among groups.

**Supplementary Table 16. Post-Hoc comparisons of TN among treatment groups (Tukey HSD Test).**

| Comparison | Mean Difference | P-value |
| --- | --- | --- |
| BE vs. C1 | -0.021 | 0.173 |
| BE vs. SC1 | -0.031 | 0.034 |
| BE vs. SC2 | -0.099 | 0 |
| C1 vs. SC1 | -0.010 | 0.667 |
| C1 vs. SC2 | -0.078 | 0 |
| SC1 vs. SC2 | -0.068 | 0 |

Note: *P*-values < 0.05 indicates significant differences.

**Supplementary Table 17. One-Way ANOVA results for TP across treatment groups**

| Source | SS | df | MS | F | P-value |
| --- | --- | --- | --- | --- | --- |
| Between Treatments | 0.008 | 3 | 0.003 | 5.593 | 0.023 |
| Within Treatments | 0.004 | 8 | 0 |  |  |
| Total | 0.012 | 11 |  |  |  |

Note: *P*-values < 0.05 indicates significant differences among groups.

**Supplementary Table 18. Post-Hoc comparisons of TP among treatment groups (Tukey HSD Test).**

| Comparison | Mean Difference | P-value |
| --- | --- | --- |
| BE vs. C1 | -0.008 | 0.968 |
| BE vs. SC1 | -0.048 | 0.099 |
| BE vs. SC2 | -0.061 | 0.037 |
| C1 vs. SC1 | -0.040 | 0.185 |
| C1 vs. SC2 | -0.053 | 0.070 |
| SC1 vs. SC2 | -0.012 | 0.897 |

Note: *P*-values < 0.05 indicates significant differences.

**Supplementary Table 19. One-Way ANOVA results for TK across treatment groups**

| Source | SS | df | MS | F | P-value |
| --- | --- | --- | --- | --- | --- |
| Between Treatments | 0.091 | 3 | 0.030 | 3.243 | 0.081 |
| Within Treatments | 0.075 | 8 | 0.009 |  |  |
| Total | 0.166 | 11 |  |  |  |

Note: *P*-values < 0.05 indicates significant differences among groups.

**Supplementary Table 20. Post-Hoc comparisons of TK among treatment groups (Tukey HSD Test).**

| Comparison | Mean Difference | P-value |
| --- | --- | --- |
| BE vs. C1 | 0.200 | 0.128 |
| BE vs. SC1 | 0.056 | 0.893 |
| BE vs. SC2 | -0.024 | 0.989 |
| C1 vs. SC1 | -0.144 | 0.328 |
| C1 vs. SC2 | -0.224 | 0.083 |
| SC1 vs. SC2 | -0.080 | 0.747 |

Note: *P*-values < 0.05 indicates significant differences.

**Supplementary Table 21. One-Way ANOVA results for HN across treatment groups**

| Source | SS | df | MS | F | P-value |
| --- | --- | --- | --- | --- | --- |
| Between Treatments | 4181.157 | 3 | 1393.719 | 170.59 | 0 |
| Within Treatments | 65.360 | 8 | 8.170 |  |  |
| Total | 4246.517 | 11 |  |  |  |

Note: *P*-values < 0.05 indicates significant differences among groups.

**Supplementary Table 22. Post-Hoc comparisons of HN among treatment groups (Tukey HSD Test).**

| Comparison | Mean Difference | P-value |
| --- | --- | --- |
| BE vs. C1 | 14.533 | 0.001 |
| BE vs. SC1 | -12.533 | 0.003 |
| BE vs. SC2 | -36.333 | 0 |
| C1 vs. SC1 | -27.067 | 0 |
| C1 vs. SC2 | -50.867 | 0 |
| SC1 vs. SC2 | -23.800 | 0 |

Note: *P*-values < 0.05 indicates significant differences.

**Supplementary Table 23. One-Way ANOVA results for Ava-P across treatment groups**

| Source | SS | df | MS | F | P-value |
| --- | --- | --- | --- | --- | --- |
| Between Treatments | 174.673 | 3 | 58.224 | 15.871 | 0.001 |
| Within Treatments | 29.348 | 8 | 3.669 |  |  |
| Total | 204.022 | 11 |  |  |  |

Note: *P*-values < 0.05 indicates significant differences among groups.

**Supplementary Table 24. Post-Hoc comparisons of Ava-P among treatment groups (Tukey HSD Test).**

| Comparison | Mean Difference | P-value |
| --- | --- | --- |
| BE vs. C1 | 2.107 | 0.562 |
| BE vs. SC1 | -5.270 | 0.040 |
| BE vs. SC2 | -7.310 | 0.007 |
| C1 vs. SC1 | -7.377 | 0.007 |
| C1 vs. SC2 | -9.417 | 0.001 |
| SC1 vs. SC2 | -2.040 | 0.585 |

Note: *P*-values < 0.05 indicates significant differences.

**Supplementary Table 25. One-Way ANOVA results for Ava-K across treatment groups**

| Source | SS | df | MS | F | P-value |
| --- | --- | --- | --- | --- | --- |
| Between Treatments | 3176.209 | 3 | 1058.736 | 13.471 | 0.002 |
| Within Treatments | 628.761 | 8 | 78.595 |  |  |
| Total | 3804.97 | 11 |  |  |  |

Note: *P*-values < 0.05 indicates significant differences among groups.

**Supplementary Table 26. Post-Hoc comparisons of Ava-K among treatment groups (Tukey HSD Test).**

| Comparison | Mean Difference | P-value |
| --- | --- | --- |
| BE vs. C1 | 8.890 | 0.628 |
| BE vs. SC1 | -24.163 | 0.041 |
| BE vs. SC2 | -30.220 | 0.013 |
| C1 vs. SC1 | -33.053 | 0.008 |
| C1 vs. SC2 | -39.110 | 0.003 |
| SC1 vs. SC2 | -6.057 | 0.836 |

Note: *P*-values < 0.05 indicates significant differences.

**Supplementary Table 27.** Soil enzyme activities before and after treatment.

| Treatments | S-SC | S-UE | S-ACP |
| --- | --- | --- | --- |
| BE-1 | 4.420 | 235.960 | 602.280 |
| BE-2 | 4.540 | 249.200 | 540.260 |
| BE-3 | 4.360 | 219.200 | 569.770 |
| C1-1 | 4.300 | 242.710 | 594.960 |
| C1-2 | 4.440 | 238.210 | 570.580 |
| C1-3 | 4.720 | 251.730 | 543.270 |
| SC1-1 | 5.060 | 305.800 | 757.700 |
| SC1-2 | 5.140 | 310.300 | 703.630 |
| SC1-3 | 5.280 | 294.540 | 738.020 |
| SC2-1 | 5.570 | 373.120 | 920.160 |
| SC2-2 | 5.680 | 400.130 | 967.470 |
| SC2-3 | 5.810 | 396.910 | 907.150 |

Note: S-SC, soil sucrase; S-UE, soil urease; S-ACP, soil acid phosphatase

**Supplementary Table 28. Shapiro-Wilk test results for soil enzyme activities before and after treatment.**

| Variable | Test Method | Statistic | P-value |
| --- | --- | --- | --- |
| S-SC | Shapiro-Wilk | 0.899 | 0.155 |
| S-UE | Shapiro-Wilk | 0.864 | 0.054 |
| S-ACP | Shapiro-Wilk | 0.862 | 0.052 |

Note: *P*-values < 0.05 indicate deviation from normality. S-SC, soil sucrase; S-UE, soil urease; S-ACP, soil acid phosphatase

**Supplementary Table 29. Levene’s test results for variance homogeneity across soil enzyme activities before and after treatment.**

| Variable | Test Method | Statistic | P-value |
| --- | --- | --- | --- |
| S-SC | Levene’s | 1.063 | 0.417 |
| S-UE | Levene’s | 1.047 | 0.423 |
| S-ACP | Levene’s | 0.103 | 0.956 |

Note: *P*-values < 0.05 indicates significant heterogeneity of variance. S-SC, soil sucrase; S-UE, soil urease; S-ACP, soil acid phosphatase

**Supplementary Table 30. One-Way ANOVA results for soil sucrase across treatment groups**

| Source | SS | df | MS | F | P-value |
| --- | --- | --- | --- | --- | --- |
| Between Treatments | 3.184 | 3 | 1.061 | 52.435 | 0 |
| Within Treatments | 0.162 | 8 | 0.02 |  |  |
| Total | 3.346 | 11 |  |  |  |

Note: *P*-values < 0.05 indicates significant differences among groups.

**Supplementary Table 31. Post-Hoc comparisons of soil sucrase among treatment groups (Tukey HSD Test).**

| Comparison | Mean Difference | P-value |
| --- | --- | --- |
| BE vs. C1 | -0.047 | 0.977 |
| BE vs. SC1 | -0.720 | 0.001 |
| BE vs. SC2 | -1.247 | 0 |
| C1 vs. SC1 | -0.673 | 0.002 |
| C1 vs. SC2 | -1.200 | 0 |
| SC1 vs. SC2 | -0.527 | 0.008 |

Note: *P*-values < 0.05 indicates significant differences.

**Supplementary Table 32. One-Way ANOVA results for soil urease across treatment groups**

| Source | SS | df | MS | F | P-value |
| --- | --- | --- | --- | --- | --- |
| Between Treatments | 45897.289 | 3 | 15299.096 | 109.872 | 0 |
| Within Treatments | 1113.961 | 8 | 139.245 |  |  |
| Total | 47011.251 | 11 |  |  |  |

Note: *P*-values < 0.05 indicates significant differences among groups.

**Supplementary Table 33. Post-Hoc comparisons of soil urease among treatment groups (Tukey HSD Test).**

| Comparison | Mean Difference | P-value |
| --- | --- | --- |
| BE vs. C1 | -9.430 | 0.765 |
| BE vs. SC1 | -68.760 | 0 |
| BE vs. SC2 | -155.267 | 0 |
| C1 vs. SC1 | -59.330 | 0.001 |
| C1 vs. SC2 | -145.837 | 0 |
| SC1 vs. SC2 | -86.507 | 0 |

Note: *P*-values < 0.05 indicates significant differences.

**Supplementary Table 34. One-Way ANOVA results for soil acid phosphatase across treatment groups**

| Source | SS | df | MS | F | P-value |
| --- | --- | --- | --- | --- | --- |
| Between Treatments | 265288.227 | 3 | 88429.409 | 104.414 | 0 |
| Within Treatments | 6775.278 | 8 | 846.910 |  |  |
| Total | 272063.506 | 11 |  |  |  |

Note: *P*-values < 0.05 indicates significant differences among groups.

**Supplementary Table 35. Post-Hoc comparisons of soil acid phosphatase among treatment groups (Tukey HSD Test).**

| Comparison | Mean Difference | P-value |
| --- | --- | --- |
| BE vs. C1 | 1.167 | 1 |
| BE vs. SC1 | -162.347 | 0.001 |
| BE vs. SC2 | -360.823 | 0 |
| C1 vs. SC1 | -163.513 | 0.001 |
| C1 vs. SC2 | -361.990 | 0 |
| SC1 vs. SC2 | -198.477 | 0 |

Note: *P*-values < 0.05 indicates significant differences.

**Supplementary Table 36.** The name, sequence of the primer pairs.

| Primer name | Primers sequence ( 5'-3' ) | Application |
| --- | --- | --- |
| 799A-F | AACMGGATTAGATACCCKG-3 | bacterial 16S rRNA gene V4–V5 hypervariable region |
| 1193-R | ACGTCATCCCCACCTTCC |  |
| ITS1-F | CTTGGTCATTTAGAGGAAGTAA | fungal internal transcribed spacer (ITS1) region |
| ITS1-R | GCTGCGTTCTTCATCGATGC |  |

**Supplementary Table 37.** Yield and content of tetrahydropalmatine and protopine in *C. yanhusuo*.

| Treatments | Yield(Kg/ha) | Tetrahydropalmatine(%) | Protopine(%) |
| --- | --- | --- | --- |
| C1-1 | 9000 | 0.215 | 0.938 |
| C1-2 | 8600 | 0.214 | 0.901 |
| C1-3 | 9700 | 0.223 | 0.929 |
| SC1-1 | 12100 | 0.233 | 0.985 |
| SC1-2 | 12900 | 0.226 | 1.178 |
| SC1-3 | 12500 | 0.234 | 1.080 |
| SC2-1 | 14100 | 0.261 | 1.267 |
| SC2-2 | 14600 | 0.284 | 1.366 |
| SC2-3 | 13900 | 0.281 | 1.269 |

**Supplementary Table 38. Shapiro-Wilk test results for yield and content of tetrahydropalmatine and protopine in *C. yanhusuo*.**

| Variable | Test Method | Statistic | P-value |
| --- | --- | --- | --- |
| yield | Shapiro-Wilk | 0.891 | 0.207 |
| tetrahydropalmatine | Shapiro-Wilk | 0.850 | 0.074 |
| protopine | Shapiro-Wilk | 0.903 | 0.272 |

Note: *P*-values < 0.05 indicate deviation from normality.

**Supplementary Table 39. Levene’s test results for yield and content of tetrahydropalmatine and protopine in *C. yanhusuo*.**

| Variable | Test Method | Statistic | P-value |
| --- | --- | --- | --- |
| yield | Levene’s | 0.364 | 0.709 |
| tetrahydropalmatine | Levene’s | 4.403 | 0.067 |
| protopine | Levene’s | 1.656 | 0.268 |

Note: *P*-values < 0.05 indicates significant heterogeneity of variance.

**Supplementary Table 40. One-Way ANOVA results for yield across treatment groups**

| Source | SS | df | MS | F | P-value |
| --- | --- | --- | --- | --- | --- |
| Between Treatments | 40460000 | 2 | 20230000 | 101.15 | 0 |
| Within Treatments | 1200000 | 6 | 200000 |  |  |
| Total | 41660000 | 8 |  |  |  |

Note: *P*-values < 0.05 indicates significant differences among groups.

**Supplementary Table 41. Post-Hoc comparisons of yield among treatment groups (Tukey HSD Test).**

| Comparison | Mean Difference | P-value |
| --- | --- | --- |
| C1 vs. SC1 | -3400 | 0 |
| C1 vs. SC2 | -5100 | 0 |
| SC1 vs. SC2 | -1700 | 0.008 |

Note: *P*-values < 0.05 indicates significant differences.

**Supplementary Table 42. One-Way ANOVA results for content of tetrahydropalmatine across treatment groups**

| Source | SS | df | MS | F | P-value |
| --- | --- | --- | --- | --- | --- |
| Between Treatments | 0.006 | 2 | 0.003 | 41.441 | 0 |
| Within Treatments | 0 | 6 | 0 |  |  |
| Total | 0.006 | 8 |  |  |  |

Note: *P*-values < 0.05 indicates significant differences among groups.

**Supplementary Table 43. Post-Hoc comparisons of content of tetrahydropalmatine among treatment groups (Tukey HSD Test).**

| Comparison | Mean Difference | P-value |
| --- | --- | --- |
| C1 vs. SC1 | -0.014 | 0.181 |
| C1 vs. SC2 | -0.058 | 0 |
| SC1 vs. SC2 | -0.044 | 0.001 |

Note: *P*-values < 0.05 indicates significant differences.

**Supplementary Table 44. One-Way ANOVA results for content of protopine across treatment groups**

| Source | SS | df | MS | F | P-value |
| --- | --- | --- | --- | --- | --- |
| Between Treatments | 0.216 | 2 | 0.108 | 25.164 | 0.001 |
| Within Treatments | 0.026 | 6 | 0.004 |  |  |
| Total | 0.242 | 8 |  |  |  |

Note: *P*-values < 0.05 indicates significant differences among groups.

**Supplementary Table 45. Post-Hoc comparisons of content of protopine among treatment groups (Tukey HSD Test).**

| Comparison | Mean Difference | P-value |
| --- | --- | --- |
| C1 vs. SC1 | -0.158 | 0.057 |
| C1 vs. SC2 | -0.378 | 0.001 |
| SC1 vs. SC2 | -0.220 | 0.015 |

Note: *P*-values < 0.05 indicates significant differences.

**Supplementary Table 46**. High-throughput results for bacteria and fungi under different cropping practices.

| Sample | Bacteria | | Fungi | |
| --- | --- | --- | --- | --- |
|  | Reads | OTU | Reads | OTU |
| C1_BS_1 | 67458 | 2852 | 94497 | 825 |
| C1_BS_2 | 77924 | 3425 | 85730 | 661 |
| C1_BS_3 | 69231 | 3195 | 102166 | 928 |
| C1_R_1 | 62580 | 581 | 88083 | 160 |
| C1_R_2 | 67146 | 741 | 61535 | 219 |
| C1_R_3 | 63991 | 608 | 88019 | 179 |
| C1_RS_1 | 71111 | 2895 | 41584 | 664 |
| C1_RS_2 | 63138 | 2436 | 78994 | 508 |
| C1_RS_3 | 56958 | 2518 | 56920 | 723 |
| SC1_BS_1 | 68866 | 3399 | 97535 | 1080 |
| SC1_BS_2 | 64171 | 3158 | 93219 | 1067 |
| SC1_BS_3 | 64378 | 3075 | 95804 | 1081 |
| SC1_R_1 | 67770 | 874 | 86390 | 184 |
| SC1_R_2 | 57178 | 643 | 89000 | 130 |
| SC1_R_3 | 65371 | 520 | 73655 | 127 |
| SC1_RS_1 | 57068 | 2772 | 69772 | 704 |
| SC1_RS_2 | 61915 | 2501 | 91748 | 696 |
| SC1_RS_3 | 58103 | 2489 | 63603 | 744 |
| SC2_BS_1 | 72886 | 3519 | 96998 | 1040 |
| SC2_BS_2 | 77462 | 3501 | 110780 | 1061 |
| SC2_BS_3 | 71005 | 3523 | 102496 | 1069 |
| SC2_R_1 | 75526 | 980 | 99350 | 264 |
| SC2_R_2 | 75936 | 1314 | 89111 | 321 |
| SC2_R_3 | 72705 | 1104 | 84337 | 196 |
| SC2_RS_1 | 66194 | 3125 | 106799 | 957 |
| SC2_RS_2 | 72716 | 3256 | 93724 | 945 |
| SC2_RS_3 | 67117 | 3358 | 99596 | 893 |

**Supplementary Table 47**. Topological properties of bacterial and fungal co-occurrence networks from different cropping practices.

| Network Indexes | Bacteria | | | Fungi | | |
| --- | --- | --- | --- | --- | --- | --- |
|  | C1 | SC1 | SC2 | C1 | SC1 | SC2 |
| Total nodes | 149 | 156 | 194 | 82 | 91 | 110 |
| Total links | 894 | 1310 | 1711 | 330 | 421 | 571 |
| Modularity | 0.44 | 0.49 | 0.53 | 0.45 | 0.47 | 0.49 |
| Average clustering coefficient | 0.48 | 0.51 | 0.55 | 0.48 | 0.52 | 0.61 |
| Average path length | 3.34 | 3 | 2.84 | 3.71 | 3.4 | 3.3 |
| Density | 0.08 | 0.09 | 0.1 | 0.09 | 0.09 | 0.1 |

**Supplementary Table 48**. Data of soil nutrients, microbial communities, and the yield and quality of *C. yanhusuo*. used in SEM.

| Treatments | HN | TN | SOM | Yield | Tetrahydropalmatine | Protopine | Bacteria Community composition | Fungi Community composition |
| --- | --- | --- | --- | --- | --- | --- | --- | --- |
| C1_BS_1 | 196.00 | 1.688 | 11.84 | 9000 | 0.215 | 0.938 | -0.250 | -0.460 |
| C1_BS_2 | 197.40 | 1.663 | 11.37 | 8600 | 0.214 | 0.901 | -0.211 | -0.488 |
| C1_BS_3 | 194.60 | 1.688 | 13.31 | 9700 | 0.223 | 0.929 | -0.193 | -0.354 |
| SC1_BS_1 | 222.60 | 1.680 | 16.30 | 12100 | 0.233 | 0.985 | 0.084 | 0.221 |
| SC1_BS_2 | 221.20 | 1.690 | 14.32 | 12900 | 0.226 | 1.178 | 0.079 | 0.228 |
| SC1_BS_3 | 225.40 | 1.700 | 13.08 | 12500 | 0.234 | 1.080 | 0.094 | 0.225 |
| SC2_BS_1 | 247.80 | 1.758 | 15.83 | 14100 | 0.261 | 1.267 | 0.010 | 0.150 |
| SC2_BS_2 | 242.20 | 1.752 | 17.54 | 14600 | 0.284 | 1.366 | 0.030 | 0.137 |
| SC2_BS_3 | 250.60 | 1.764 | 15.95 | 13900 | 0.281 | 1.269 | 0.028 | 0.166 |
| C1_R_1 | 196.00 | 1.688 | 11.84 | 9000 | 0.215 | 0.938 | -0.094 | -0.110 |
| C1_R_2 | 197.40 | 1.663 | 11.37 | 8600 | 0.214 | 0.901 | -0.130 | -0.112 |
| C1_R_3 | 194.60 | 1.688 | 13.31 | 9700 | 0.223 | 0.929 | -0.104 | -0.105 |
| SC1_R_1 | 222.60 | 1.680 | 16.30 | 12100 | 0.233 | 0.985 | 0.209 | 0.076 |
| SC1_R_2 | 221.20 | 1.690 | 14.32 | 12900 | 0.226 | 1.178 | 0.215 | 0.062 |
| SC1_R_3 | 225.40 | 1.700 | 13.08 | 12500 | 0.234 | 1.080 | 0.238 | 0.095 |
| SC2_R_1 | 247.80 | 1.758 | 15.83 | 14100 | 0.261 | 1.267 | -0.087 | -0.317 |
| SC2_R_2 | 242.20 | 1.752 | 17.54 | 14600 | 0.284 | 1.366 | -0.032 | -0.091 |
| SC2_R_3 | 250.60 | 1.764 | 15.95 | 13900 | 0.281 | 1.269 | -0.040 | 0.027 |
| C1_RS_1 | 196.00 | 1.688 | 11.84 | 9000 | 0.215 | 0.938 | -0.246 | -0.173 |
| C1_RS_2 | 197.40 | 1.663 | 11.37 | 8600 | 0.214 | 0.901 | -0.240 | -0.275 |
| C1_RS_3 | 194.60 | 1.688 | 13.31 | 9700 | 0.223 | 0.929 | -0.240 | -0.174 |
| SC1_RS_1 | 222.60 | 1.680 | 16.30 | 12100 | 0.233 | 0.985 | 0.221 | 0.188 |
| SC1_RS_2 | 221.20 | 1.690 | 14.32 | 12900 | 0.226 | 1.178 | 0.237 | 0.234 |
| SC1_RS_3 | 225.40 | 1.700 | 13.08 | 12500 | 0.234 | 1.080 | 0.212 | 0.204 |
| SC2_RS_1 | 247.80 | 1.758 | 15.83 | 14100 | 0.261 | 1.267 | 0.052 | 0.219 |
| SC2_RS_2 | 242.20 | 1.752 | 17.54 | 14600 | 0.284 | 1.366 | 0.080 | 0.200 |
| SC2_RS_3 | 250.60 | 1.764 | 15.95 | 13900 | 0.281 | 1.269 | 0.076 | 0.227 |

**Supplementary Table 49.** SEM showing the direct and indirect effects of crop rotation on soil nutrients, microbial communities, and the yield and quality of *C. yanhusuo*.

| Response | Predictor | Estimate | Std.Error | DF | Crit.Value | P-Value | Std.Estimate | Significance |
| --- | --- | --- | --- | --- | --- | --- | --- | --- |
| Yield | Protopine | 5924.62 | 1036.68 | 19 | 5.71 | 0 | 0.45 | *** |
| Yield | Tetrahydropalmatine | –19043.93 | 9040.54 | 19 | –2.10 | 0.0487 | –0.22 | * |
| Yield | HN | 37.03 | 10.80 | 19 | 3.42 | 0.0028 | 0.36 | ** |
| Yield | SOM | 269.26 | 62.00 | 19 | 4.34 | 0.0004 | 0.25 | *** |
| Yield | TN | 5307.76 | 6656.03 | 19 | 0.80 | 0.4351 | 0.09 | - |
| Yield | Bacteria Community composition | 2330.36 | 669.29 | 19 | 2.70 | 0.0141 | 0.18 | * |
| Yield | Fungi Community composition | 662.67 | 480.50 | 19 | 0.81 | 0.8000 | 0.06 | - |
| Protopine | Tetrahydropalmatine | 1.14 | 2.83 | 20 | 0.59 | 0.5624 | 0.18 | - |
| Protopine | Tetrahydropalmatine | 0.0006 | 0.0023 | 20 | 0.26 | 0.7943 | 0.08 | - |
| Protopine | TN | 2.79 | 1.30 | 20 | 2.16 | 0.0431 | 0.61 | * |
| Protopine | SOM | 0.0034 | 0.01 | 20 | 0.26 | 0.7989 | 0.04 | - |
| Protopine | Bacteria Community composition | 0.23 | 0.18 | 20 | 1.30 | 0.2115 | 0.23 | - |
| Protopine | Fungi Community composition | –0.02 | 0.10 | 20 | –0.23 | 0.8232 | –0.03 | - |
| Tetrahydropalmatine | HN | 0.0003 | 0.0002 | 22 | 1.19 | 0.2483 | 0.24 | - |
| Tetrahydropalmatine | SOM | 0.0041 | 0.0012 | 22 | 3.47 | 0.0022 | 0.33 | ** |
| Tetrahydropalmatine | TN | 0.36 | 0.12 | 22 | 2.97 | 0.007 | 0.51 | ** |
| Tetrahydropalmatine | Bacteria Community composition | –0.02 | 0.01 | 22 | –1.16 | 0.2569 | –0.11 | - |
| SOM | Bacteria Community composition | 1.25 | 3.70 | 24 | 0.34 | 0.7381 | 1.00 | - |
| SOM | Fungi Community composition | 4.35 | 2.63 | 24 | 1.64 | 0.1639 | 0.48 | - |
| TN | SOM | 0.01 | 0.0029 | 23 | 4.76 | 0.0001 | 0.78 | *** |
| TN | Bacteria Community composition | –0.10 | 0.05 | 23 | –1.87 | 0.0741 | 0.45 | - |
| TN | Fungi Community composition | 0.05 | 0.04 | 23 | 1.15 | 0.262 | 0.29 | - |
| HN | SOM | 1.33 | 0.98 | 22 | 1.356 | 0.1879 | 0.13 | - |
| HN | TN | 427.28 | 49.71 | 22 | 8.59 | 0 | 0.73 | *** |
| HN | Bacteria Community composition | 45.14 | 13.54 | 22 | 3.36 | 0.003 | 0.35 | ** |
| HN | Fungi Community composition | –0.75 | 9.77 | 22 | –0.08 | 0.9397 | –0.008 | - |
| Bacteria Community composition | Fungi Community composition | 0.59 | 0.08 | 25 | 7.30 | 0 | 0.82 | *** |


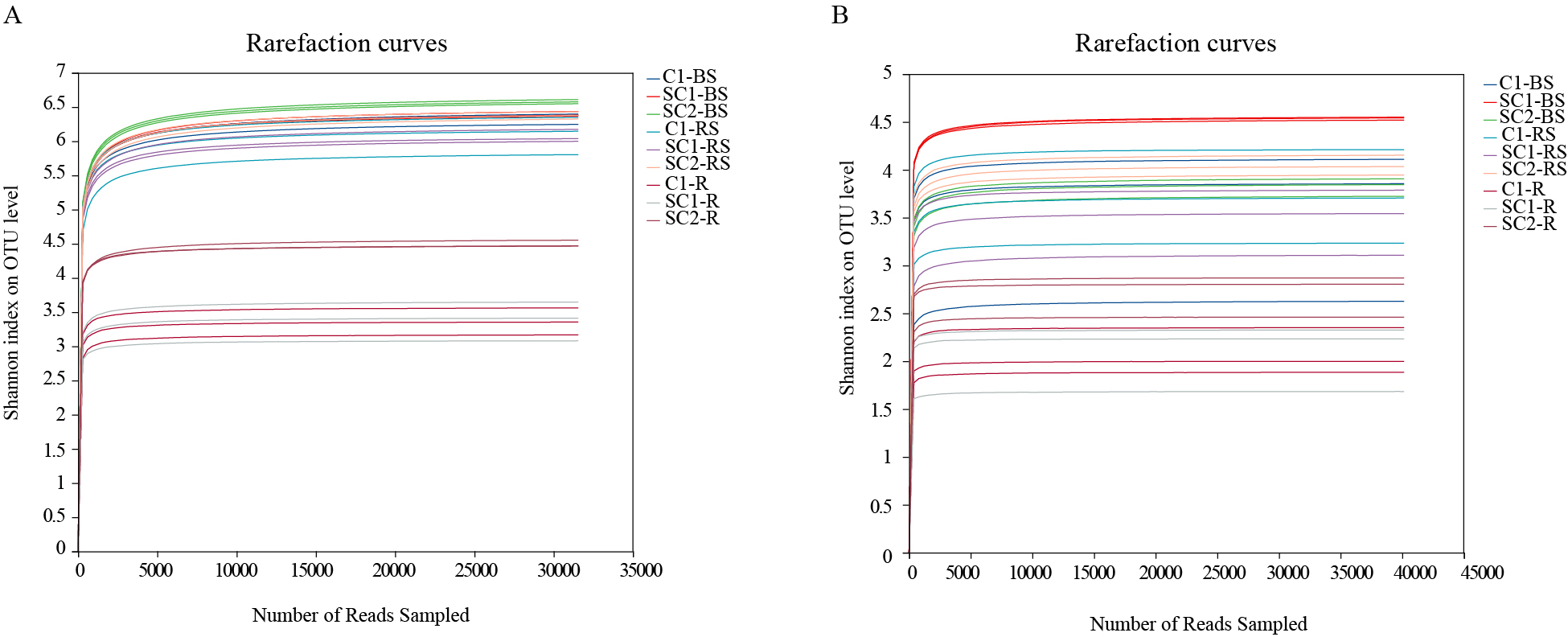


**Supplementary Figure 1.** Rarefaction curves of samples under different cropping practices. (A) Bacteria, (B) Fungi.


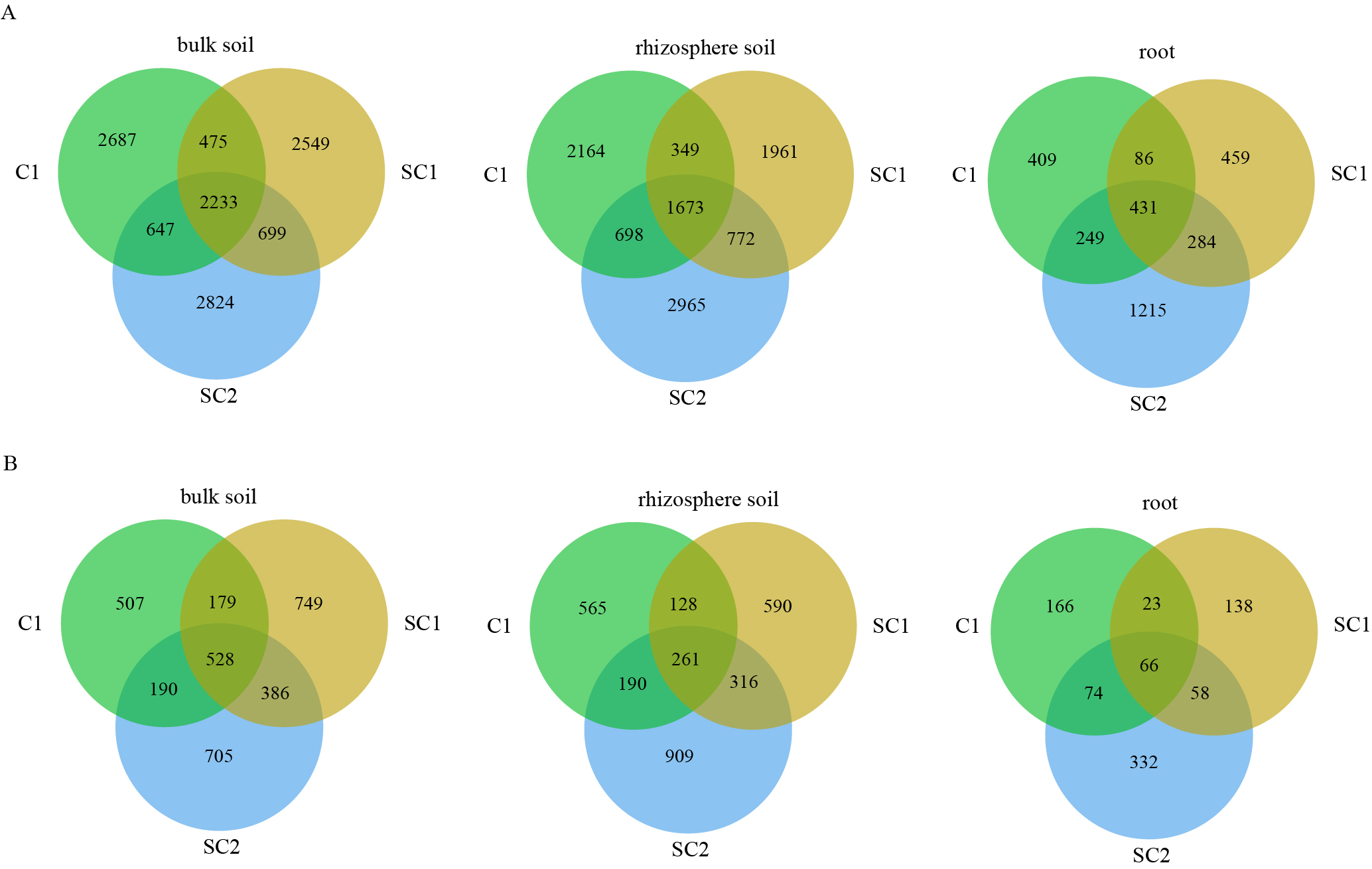


**Supplementary Figure 2.** Venn diagram of microbial communities of different cropping practices. (A) The number of bacterial OTUs. (B) The number of fungal OTUs.


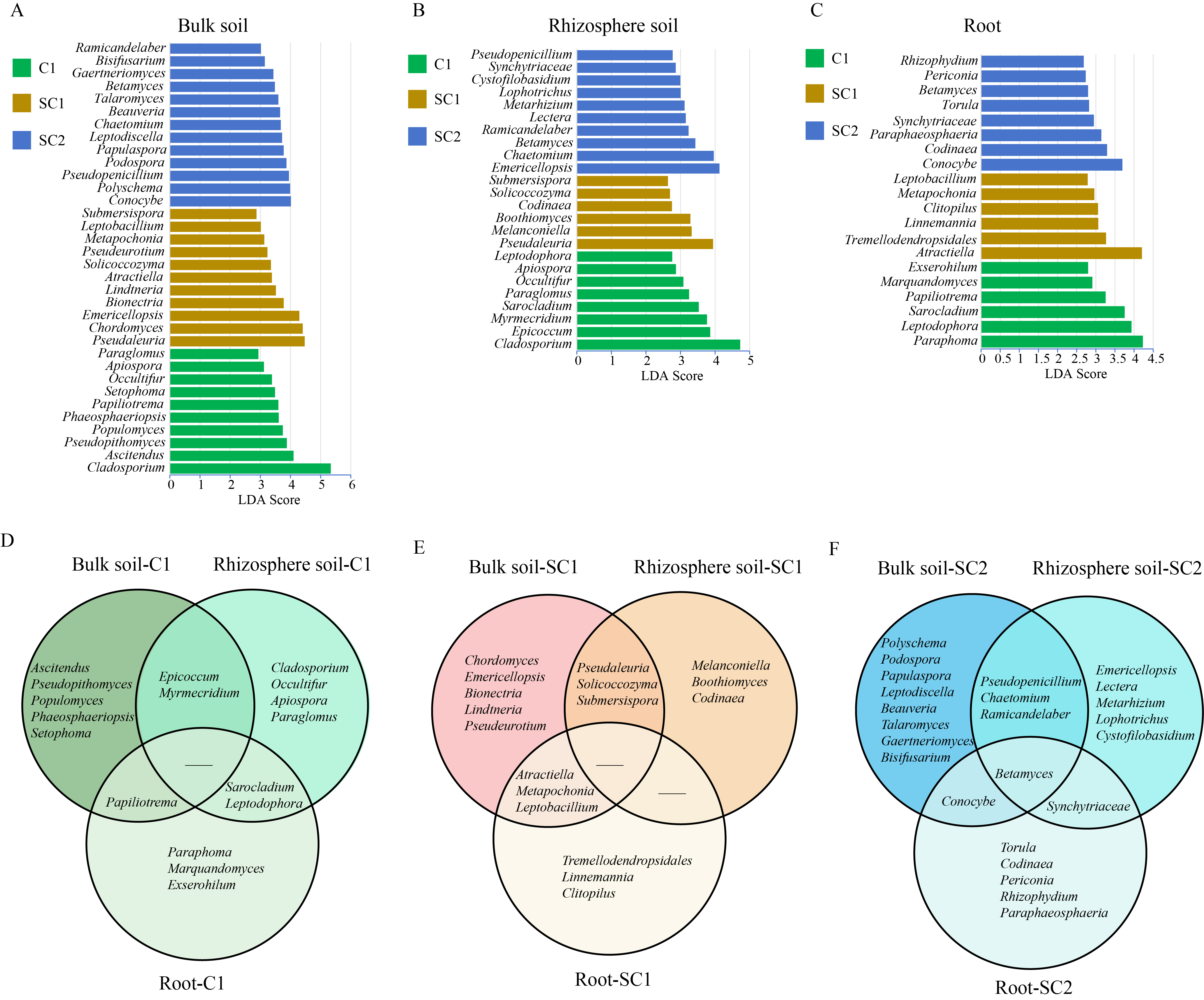


**Supplementary Figure 3.** LEfSe analysis of fungal microbial community differences in different cropping practices. LDA based on the level of fungal microbial genera showed the microbial classes that caused significant differences between different treatments in bulk soil (A), rhizosphere soil (B), and roots (C). The green, yellow, and blue indicate that the taxa are in the C1, SC1, and SC2 groups, respectively. Veen analysis of fungal communities in bulk soil, rhizosphere soil and roots in the different cropping systems. Fungal community in C1 (D), SC1 (E), and SC2 (F).


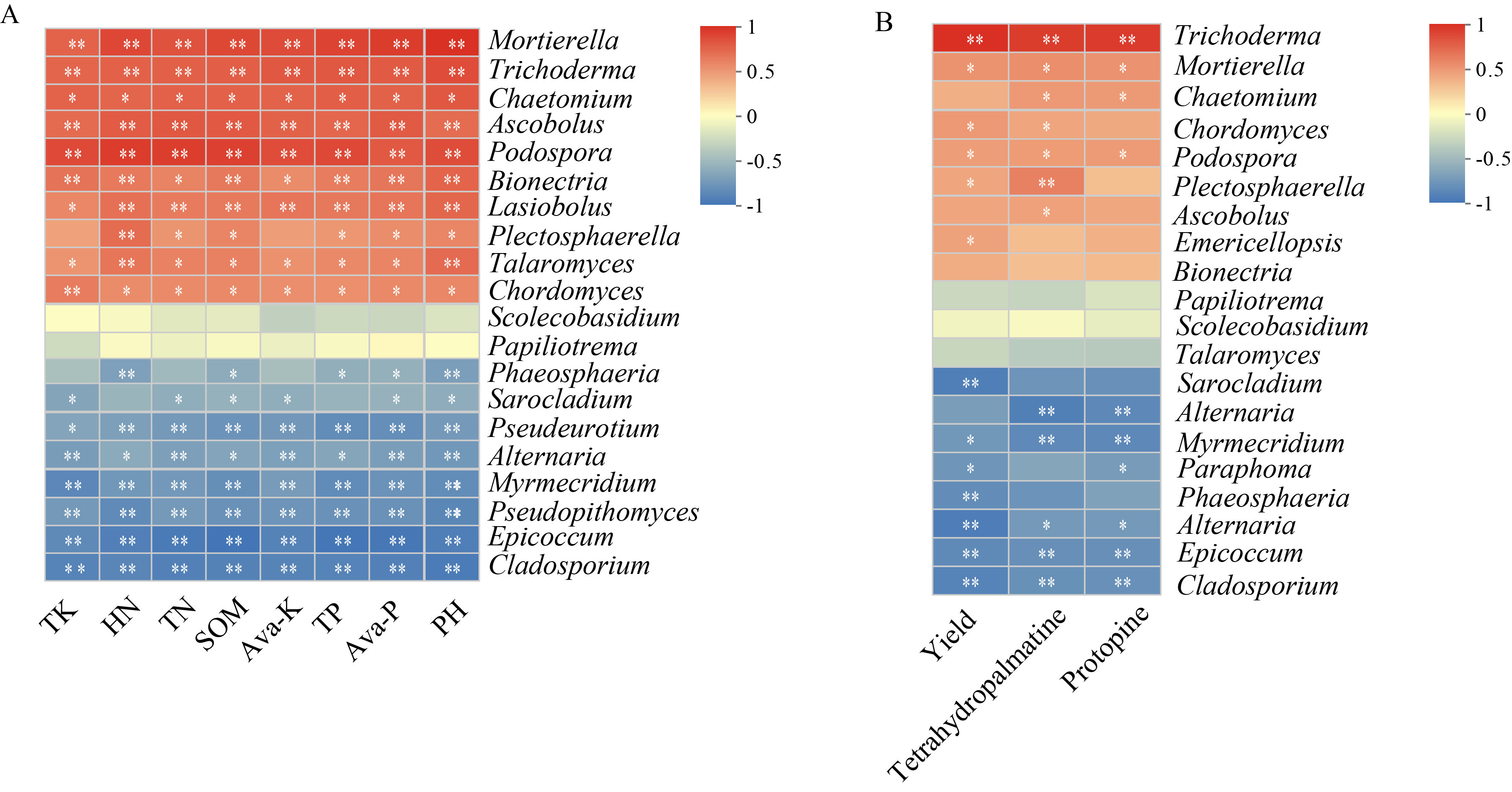


**Supplementary Figure 4.** Relationship between microorganisms, soil factors, yield and active ingredients of *C. yanhusuo*. Correlation heatmap of fungal genera with soil factors (A) and with active ingredients and yield of *C. yanhusuo* (B). **P* < 0.05; ***P* < 0.01.


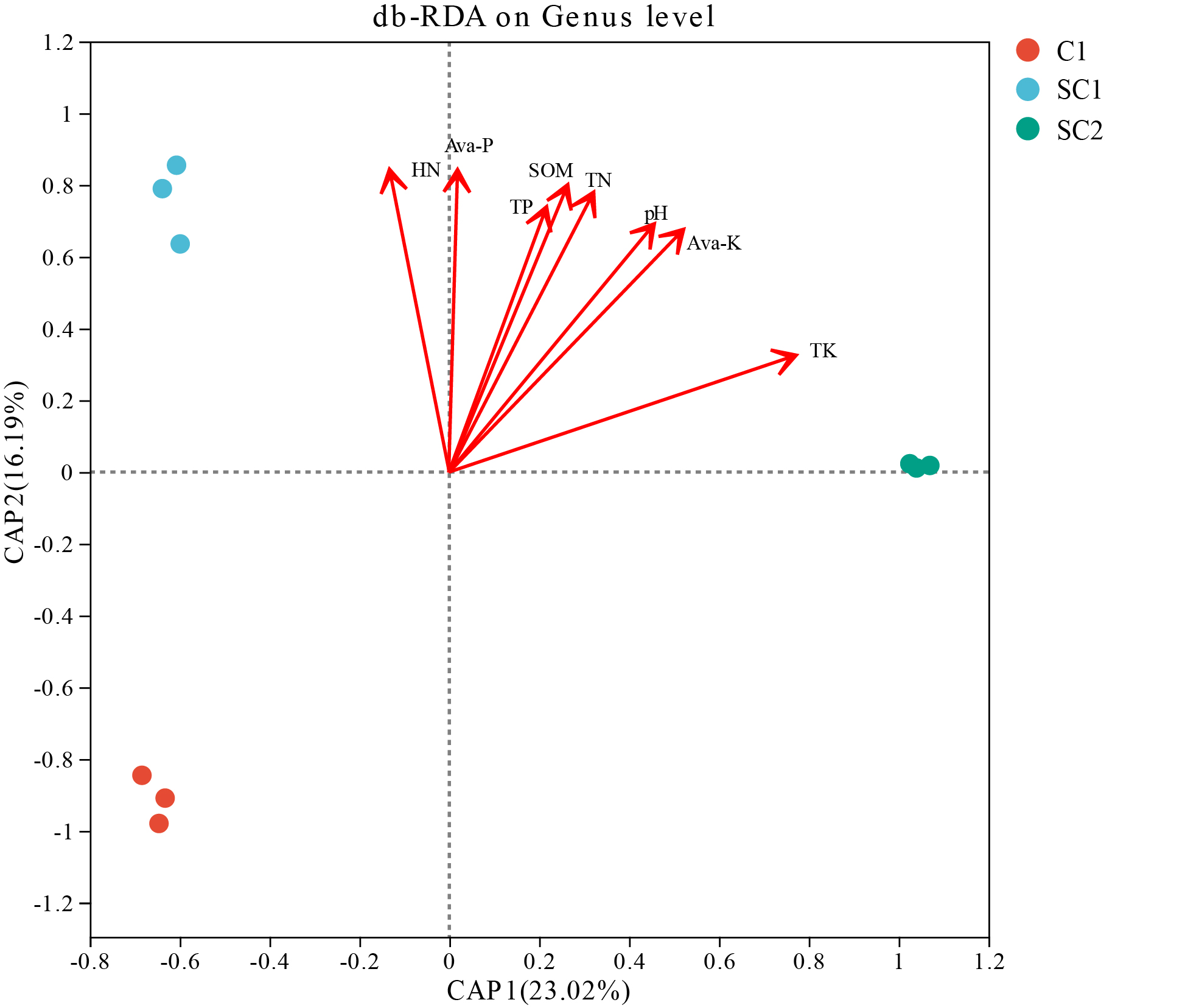


**Supplementary Figure 5.** Distance-based Redundancy Analysis on soil physicochemical properties and fungal microbial communities of *C. yanhusuo*.
